# Canine *CNGA3* Gene Mutations Provide Novel Insights into Human Achromatopsia-Associated Channelopathies and Treatment

**DOI:** 10.1371/journal.pone.0138943

**Published:** 2015-09-25

**Authors:** Naoto Tanaka, Emily V. Dutrow, Keiko Miyadera, Lucie Delemotte, Christopher M. MacDermaid, Shelby L. Reinstein, William R. Crumley, Christopher J. Dixon, Margret L. Casal, Michael L. Klein, Gustavo D. Aguirre, Jacqueline C. Tanaka, Karina E. Guziewicz

**Affiliations:** 1 Department of Biology, College of Science and Technology, Temple University, Philadelphia, Pennsylvania, United States of America; 2 Department of Clinical Studies-Philadelphia, School of Veterinary Medicine, University of Pennsylvania, Philadelphia, Pennsylvania, United States of America; 3 Institute for Computational Molecular Science, Temple University, Philadelphia, Pennsylvania, United States of America; 4 Veterinary Vision, Penrith, Cumbria, CA11 9FJ, United Kingdom; University of Massachusetts Medical School, UNITED STATES

## Abstract

Cyclic nucleotide-gated (CNG) ion channels are key mediators underlying signal transduction in retinal and olfactory receptors. Genetic defects in *CNGA3* and *CNGB3*, encoding two structurally related subunits of cone CNG channels, lead to achromatopsia (ACHM). ACHM is a congenital, autosomal recessive retinal disorder that manifests by cone photoreceptor dysfunction, severely reduced visual acuity, impaired or complete color blindness and photophobia. Here, we report the first canine models for *CNGA3*-associated channelopathy caused by R424W or V644del mutations in the canine *CNGA3* ortholog that accurately mimic the clinical and molecular features of human CNGA3-associated ACHM. These two spontaneous mutations exposed CNGA3 residues essential for the preservation of channel function and biogenesis. The CNGA3-R424W results in complete loss of cone function *in vivo* and channel activity confirmed by *in vitro* electrophysiology. Structural modeling and molecular dynamics (MD) simulations revealed R424-E306 salt bridge formation and its disruption with the R424W mutant. Reversal of charges in a CNGA3-R424E-E306R double mutant channel rescued cGMP-activated currents uncovering new insights into channel gating. The CNGA3-V644del affects the C-terminal leucine zipper (CLZ) domain destabilizing intersubunit interactions of the coiled-coil complex in the MD simulations; the *in vitro* experiments showed incompetent trimeric CNGA3 subunit assembly consistent with abnormal biogenesis of *in vivo* channels. These newly characterized large animal models not only provide a valuable system for studying cone-specific CNG channel function in health and disease, but also represent prime candidates for proof-of-concept studies of *CNGA3* gene replacement therapy for ACHM patients.

## Introduction

Cyclic nucleotide-gated (CNG) ion channels are important non-selective cation channels that show increased gating with cyclic nucleotides; they function as cellular transducers in signaling pathways of sensory and CNS neurons as well as in non-neuronal cells [1, 2]. Tetrameric CNG channels belong to a large voltage-gated K^**+**^ channel superfamily and are encoded by combinations of subunits from *CNGA1-CNGA4*, *CNGB1* and *CNGB3* genes. In the visual system, rod-mediated phototransduction utilizes outer segment membrane CNG channels assembled from three CNGA1 subunits and one CNGB1, whereas the cone-specific CNG channels are composed of CNGA3 and CNGB3 subunits, arranged in a proposed stoichiometric ratio of 3:1, respectively [3–5], but also see [6].

Homozygous or compound heterozygous mutations in any of five critical genes involved in the cone phototransduction cascade have been causally attributed to functional abnormalities known as complete and incomplete achromatopsia (ACHM). ACHM refers to a group of cone-selective retinal disorders characterized by loss of photopic vision, photophobia, poor visual acuity, pendular nystagmus, rod monochromacy and anomalous cone photoreceptor-mediated function caused directly by mutations in genes disrupting cone phototransduction pathway [7, 8]. Mutations associated with the CNGB3 subunit are most common in European subjects (ACHM3; OMIM# 262300) and account for 50% of all known cases worldwide, whereas the *CNGA3*-associated achromatopsia (ACHM2; OMIM# 216900) accounts for ~30% of all diagnosed cases thus far. Mutations in *GNAT2*, *PDE6C* and *PDE6H* underlie less than 2% of recognized cases [9] while recently reported deleterious variants in *ATF6* gene are responsible for rare cases of ACHM [10]. Hitherto, over 100 distinct mutations have been identified in *CNGA3* and almost 50 in the *CNGB3*, and these sequence alterations also may have an additive causative effect [11, 12]. Currently, there is no treatment available that restores cone function in achromatopsia patients.

Hereditary ion channelopathies of the central nervous and visual systems are particularly devastating, as they typically manifest in infancy and early childhood [13, 14]. Due to the relative rarity of these disorders and limited accessibility of the affected tissues, it is difficult to address most of the basic biological questions regarding the pathogenesis and molecular mechanisms of these diseases. Animal models for monogenic channelopathies, such as ACHM, have proven critical in dissecting various aspects of its complex pathophysiology and have contributed to the development of therapeutic approaches, including AAV-mediated gene replacement therapy [9, 15–22].

Disease-associated mutations in CNG channel subunits often expose residues essential for the preservation of channel functions and/or channel biogenesis. Our prior studies provided important insights into the CNG channel architecture, function and folding [23, 24]. In this study, we report the first spontaneous canine ACHM2 disease models caused by R424W and V644del mutations in the dog *CNGA3* ortholog. The CNGA3-R424W identified in German shepherd breed is analogous to the human CNGA3-R410W mutation found in both homozygous and compound heterozygous states in ACHM2 patients of European origin [25, 26]. The second naturally occurring mutation affecting Labrador retrievers, CNGA3-V644del, constitutes the first animal model for C-terminal leucine zipper (CLZ) domain, a region critical for tetrameric assembly and functional regulation of the channel [27]. We used a unique combination of bioinformatics and structural modeling with molecular dynamics simulations to identify an essential salt bridge interaction between R424 and E306 residues; electrophysiological data from the target mutations involved in this interaction support a crucial role of this salt bridge in the CNG channel function. To assess the consequences of V644del, we constructed molecular models based on the high-resolution structure of CLZ from the human CNGA3 subunit [4] and showed a loss of zipper trimerization in the V644del mutant model. Based on our findings, we propose R424W and V644del as valuable models for studying CNG channel malfunction that will help to guide the forthcoming gene augmentation therapies for ACHM2 patients.

## Materials and Methods

### Sample collection and clinical data acquisition

A 5-month-old German shepherd presented to the clinic at Ryan Veterinary Hospital (School of Veterinary Medicine, University of Pennsylvania, PA) with a history of vision loss during daylight hours. Complete ophthalmic examination was performed after pupillary dilation using slit-lamp biomicroscopy and binocular indirect ophthalmoscopy. Rod and cone mediated retinal function was assessed by electroretinography (ERG) under standard conditions [28–30]. Briefly, scotopic (dark-adapted) and photopic (light-adapted) ERGs were obtained using a RETIport ERG system (AcriVet) under standard conditions [29]. Rod responses were measured after dark-adaptation for 20 minutes with a single white flash stimulus of 0.3 Hz. After 10 minutes of light-adaptation, photopic responses were recorded using a 1 Hz single flash and 29.41 Hz flicker. Blood samples were collected as a part of a routine examination at the School of Veterinary Medicine (University of Pennsylvania, PA). Blood samples and pedigree information from affected Labrador retrievers and their non-affected relatives were collected as described previously [30]. Identical ERG recordings were performed normal sighted control dogs [30].

### Ethics statement

The testing of the dogs was done as part of the clinical assessment and examination procedures. The owners of the dogs gave permission for blood collection, and for their animals to be included in the study.

### Candidate genes study and homozygosity mapping

Genomic DNA was isolated from whole blood according to the manufacturer’s instructions (QIAamp DNA Blood Mini Kit, QIAGEN Sciences). The control panel consisted of DNA samples from 100 unrelated dogs from 18 different breeds. Cone phototransduction cascade genes *CNGB3* (NM_001003030), *CNGA3* (NM_001301112), *GNAT2* (XM_547240), *PDE6C* (XM_543934), and *PDE6H* (XM_005637111) were selected for initial screening by homozygosity mapping using intragenic and flanking SNP and microsatellites markers. A set of 9 known SNPs was genotyped for *CNGB3* (rs23520109, rs23520110, rs23474935, rs23461650, rs23461648, rs23461646, rs23461644, rs23476746, rs9019459); 7 SNPs (rs23393600, rs23393599, rs23393598, rs23393597, rs23393596, rs23393594, rs23393592) and 2 microsatellite repeats [(TG)_16_ and (AG)_16_] for *PDE6C*; 4 SNP for *GNAT2* (rs8521667, rs24378551, rs24378553, rs24328648); 4 SNPs for *PDE6H* (rs23356762, rs23410195, rs23410199, rs23341912); and 9 SNPs (rs8991406, rs22092167, rs8991405, rs22092165, rs8992756, rs22097970, rs22097969, rs22097968, rs22097967) and one STR (TCCCT)_16_ for *CNGA3*.

Gene-specific primers were designed based on Broad CanFam3.1 genome assembly (http://genome.ucsc.edu) using Primer3 software (http://bioinfo.ut.ee/primer3-0.4.0/primer3/input.htm). See S1 Table for list of primer pairs and PCR conditions used for c*CNGA3* gene screening. All amplicons were purified using ExoSAP-IT (Affymetrix), subjected to direct sequencing (3730 DNA Analyzer at NAPCore Facility at Children's Hospital of Philadelphia) and analyzed using Sequencher 5.2.4 software.

### *CNGA3 in vitro* model system

A full-length canine *CNGA3* cDNA was inserted into the pEYFPN1 expression vector and site-specific mutations were introduced as described previously [24]. All generated constructs were verified by direct sequencing. Human embryonic kidney (HEK) tsA201 cells were utilized for expression studies. For patch-clamp recordings and localization studies, cells were transfected with 1–2μg of indicated plasmid construct(s) in presence of Lipofectamine™ 2000 (Invitrogen). Post-transfection, the cells were grown at 37°C for 24-48h before being used for electrophysiological or subcellular localization analyses.

### Electrophysiology and subcellular localization studies

Inside-out patches were excised from fluorescent cells as previously described [24]. The bath and electrode solutions contained 120 mM NaCl, 2 mM EDTA, 2 mM EGTA, and 5 mM Hepes at pH 7.4. The indicated concentrations of cGMP and cAMP were added to the bath (intracellular face of the cell membrane) and 300-ms voltage pulses were applied in 20 mV steps from -80 mV to 80 mV. Net currents were obtained by subtracting the bath current from nucleotide-activated currents and analyzed as described previously [23]. For cGMP dose-response relationships, current data were averaged and normalized from multiple traces, and then normalized currents (Is) were fitted by the Hill equation:

$$I=\frac{{\text{I}}_{\max}}{1+{\left(\frac{K_{0.5}}{L}\right)}^{Nh}}$$

using computer software (Clampfit 10.0™, Table curve™, Sigmaplot™) [24]. Subcellular localization studies were performed on fixed cells as previously reported [23]. Fluorescent signals were examined under 40x and captured at 100x magnification. Unpaired t-tests were performed using QuickCalcs software (http://www.graphpad.com).

### Immunohistochemistry

For the *ex vivo* data, IHC staining was performed on 10 μm thick canine retinal cryosections that were incubated overnight at +4°C in the presence of rabbit polyclonal anti-canine CNGA3 antibody (courtesy of A. Komáromy, Michigan State University) at a concentration of 1:750. Primary antibody was visualized with Alexa Fluor 568nm goat anti-rabbit secondary antibody and cell nuclei were stained with DAPI. Slides were mounted (Gelvatol, Sigma-Aldrich) and examined by epifluorescence (Axioplan; Carl Zeiss Meditec) or confocal (Leica SP5-II, Leica) microscopy using previously published methods [31].

### CNGA3 topology prediction and R424W molecular dynamics simulations

Amino acid sequences of human (GI: 4502917) and canine (GI: 345777245) CNGA3 were used as a template for transmembrane topology prediction and MD simulations. Using PSI-Blast search (http://blast.ncbi.nlm.nih.gov/Blast.cgi), the chimeric voltage-gated K^+^ channel Kv1.2/2.1 (PDB ID: 2R9R), a molecule of solved crystal structure [32], was identified as the closest homolog of known structure of the canine CNGA3 channel subunit. Amino acid sequences were aligned using Clustal X v. 2.0.1. [33]. The S1-S6 TM domains were then assigned according to the secondary structure of Kv1.2/2.1 (GI: 16087792).

The previously reported CNGA3 homology model of transmembrane regions embedded in a fully hydrated palmitoyloleoylphosphatidylcholine (POPC) bilayer in a 120 mM NaCl solution was considered for all MD simulations [24]. The R424W point mutation was introduced with the VMD mutator plugin [34]. The MD simulations were carried out using NAMD 2.9 software [35]. Langevin dynamics was applied for temperature (300 K) and pressure (1.0 atm) control. The equations of motion were integrated using a multiple time-step algorithm [36]. Short- and long-range forces were calculated every 1 and 2 time-steps respectively, with a time step of 2.0 fs. Chemical bonds between hydrogen and heavy atoms were constrained to their equilibrium values. Long-range electrostatic forces were taken into account using the particle mesh Ewald approach [37]. The water molecules were described using the TIP3P model [38]. The simulations used the CHARMM22-CMAP force field with torsional cross-terms for the protein [39] and CHARMM27 for the phospholipids [40]. All MD simulations were performed on Owls' Nest at Temple University's supercomputing facilities.

### *In silico* modeling and molecular dynamics of V630del mutant

The CLZ domain from the human CNGA3, consisting of the complete trimeric coiled-coil region, (PDB ID: 3SWY, residues: 626–673), was identified as the closest homolog of known structure of the canine CNGA3 CLZ domain. We therefore used it as a backbone template for the wild-type and V630 deletion models. Before beginning homology modeling, the structures were relaxed using 200 steps of conjugate-gradient minimization with the generalized Born implicit solvent model implemented in the NAMD2.10 software package. This initial relaxation was used to remove any structural perturbation due to crystallization conditions and to enhance the designability of protein structures, particularly in the core positions of coiled-coils. A probabilistic design algorithm was then used to identify optimized (the most probable) side-chain rotameric states consistent with the relaxed backbone structures for both the wild-type and V630del sequences [41]. For the optimization, the total conformational energy of the sequence was constrained so that its conjugate Lagrange multiplier (an effective inverse temperature) was β = (kT)^-1^ = 0.5 mol/kcal, a typical value used in many previous reports (summarized in [42]). Side-chain rotameric states were sampled from a backbone dependent rotamer library [43]. No symmetry constraints were imposed on the side-chain rotamers for identical sites across the three helices; even still, the resulting rotameric states were nearly all symmetric due to the natural symmetry of the coil’s backbone. Molecular renders were made using the VMD molecular visualization program. Wheel diagrams were constructed using DrawCoil1.0 (http://www.grigoryanlab.org/drawcoil/).

All MD simulations involving the CLZ domain were performed in an identical manner to that of the CNGA3 MD simulations with a few modifications listed below. All simulations were performed using the NAMD 2.10 (CVS) software package. Simulations were run at a temperature of 310 K fixed using Langevin dynamics and a pressure of 1.0 atm using the Nosé-Hoover Langevin piston pressure control. Typical system setup and equilibration involved solvating the coiled-coil complex with 1 nm of water homogeneously from the surface of the protein followed by neutralization by adding 150mM NaCl using the Solvate and Autoionize plugins, respectively, provided in the VMD software package. Initial velocities were sampled from a Maxwell-Boltzmann distribution with a target temperature of 310K. Initially, 5 kcal/mol/Å constraints were placed on all heavy protein atoms followed by slow removal of the constraints over a 20 ns period to encourage the formation of favorable atomic interactions and to reduce initial large perturbations leftover from the modeling process. Trajectories were then collected for 325 ns and 430 ns for the hCNGA3 CLZ wild-type and V630del mutants, respectively.

## Results

### ACHM phenotype in the canine models

**Fig 1:**
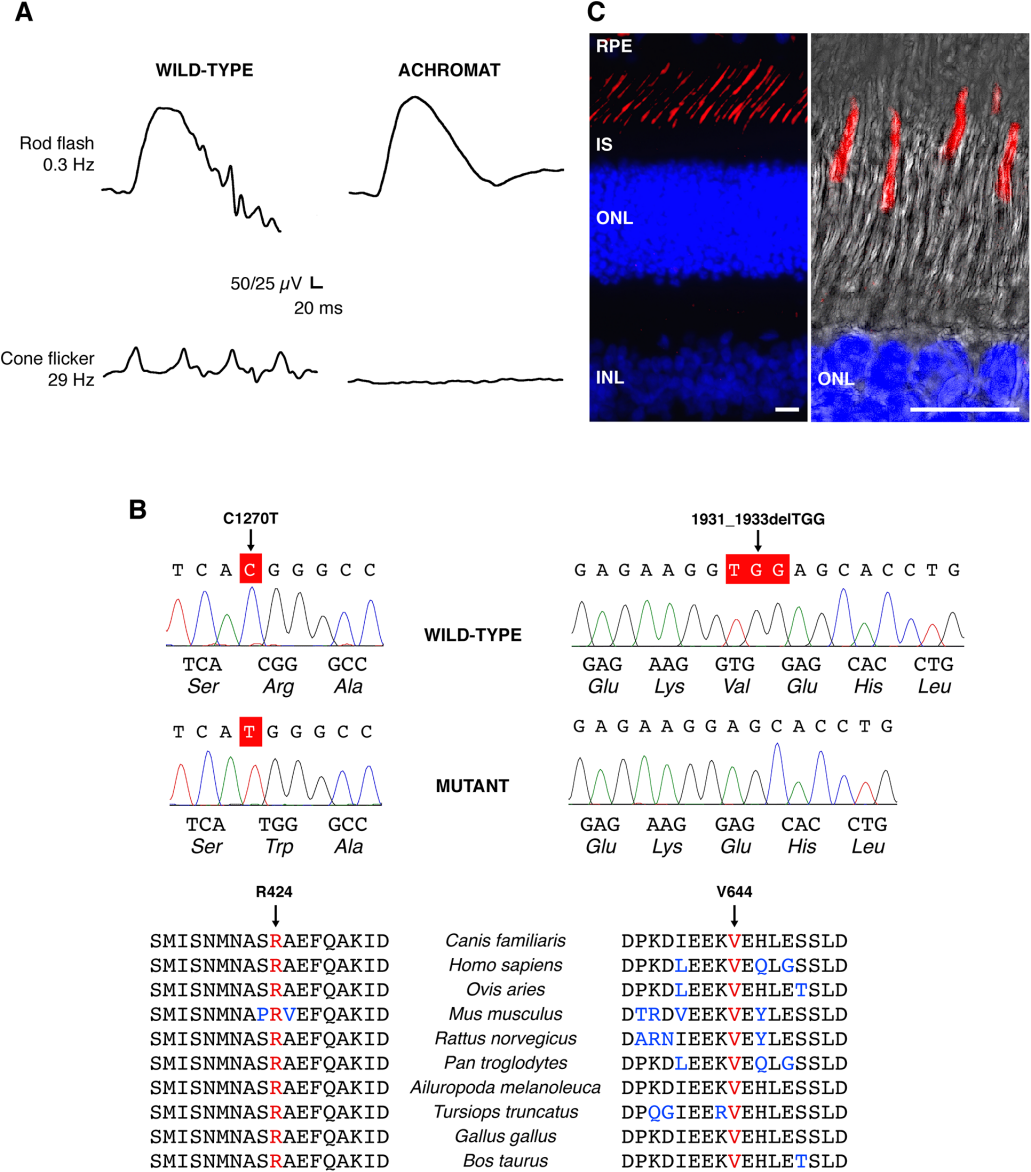
*CNGA3*-mediated channelopathy in the canine model: clinical manifestation and genetic basis. **(A)** Scotopic and photopic ERG responses obtained from a 5-month-old CNGA3-R424W-affected dog showing normal rod flash response, and absence of cone-mediated flicker responses compared to the age-matched wild-type control in ERG responses evoked under dark-adapted (rod flash, 0.3Hz) and light-adapted (cone flicker, 29 Hz) conditions. See also **S1 Fig** for behavioral vision testing under bright- and dim-light conditions. **(B)** DNA sequencing chromatograms of the wild-type and *CNGA3* spontaneous mutants showing the C1270T transition in exon 7, and the 1931_1933delTGG deletion. Both residues are highly conserved among vertebrate genomes. *Canis familiaris*—dog; *Homo sapiens*—human; *Ovis aries*—sheep; *Mus musculus*—mouse; *Rattus norvegicus*—rat; *Pan troglodytes*—chimpanzee; *Ailuropoda melanoleuca*—panda; *Tursiops truncatus*—dolphin; *Gallus gallus*—chicken; *Bos taurus*—cow. **(C)** Immunohistochemical representation of CNGA3 protein in the wild-type canine retina. CNGA3 is exclusively expressed in the outer segment of cone photoreceptor cells (labeled in red), shown also in a high-magnification confocal photomicrograph (right) representing a Z-stack of maximum projection images taken at 0.5 mm intervals created with Leica LAS-AF software (Leica Microsystems, Inc.). Cell nuclei were stained with DAPI. RPE: retinal pigment epithelium; IS: inner segment; ONL: outer nuclear layer; INL: inner nuclear layer; Scale bars: 10μm.

Young dogs from German shepherd and Labrador retriever breeds developed complete loss of photopic function at 8–10 weeks of age, soon after canine retinal development is complete [44]. Clinical ophthalmic examination was normal, but behavioral testing confirmed lack of vision during daylight hours and revealed improved visual performance under low-light conditions (**S1 Fig**). Scotopic electroretinogram (ERG) showed normal rod function, but no light-evoked cone responses under photopic conditions in affected dogs compared to the age-matched controls, indicating a complete loss of cone photoreceptor function (**Fig 1A**). Pedigree analysis in the Labrador retriever line [30] along with ophthalmic evaluations, behavioral vision testing, and ERG analyses suggested an inherited retinal disorder segregating in an autosomal recessive manner predominantly affecting cone-based vision. There was no evidence of systemic involvement or other neurological abnormalities in any of the affected dogs.

### Two distinct mutations in *CNGA3* establish first canine models of human ACHM2-associated channelopathies

Phenotype-based candidate gene analysis combined with homozygosity mapping excluded *CNGB3* (OMIM# 605080), *GNAT2* (OMIM# 139340), *PDE6C* (OMIM# 600827) and *PDE6H* (OMIM# 601190) loci from causative association in both breeds. Direct sequencing of all exons and flanking intronic sites of *CNGA3* (OMIM# 600053) revealed a missense mutation in exon 7 (c.C1270T/p.R424W) in the German shepherd breed and a 3nt deletion in exon 7 (c.1931_1933delTGG/p.V644del) in Labrador retrievers (**Fig 1B**). In the homozygous affected German shepherd, the C1270T transition results in substitution of an evolutionarily conserved basic arginine (Arg/R) at the amino acid position 424 for an aromatic, hydrophobic tryptophan (Trp/W) residue, the largest amino acid. The homozygous 1931_1933delTGG mutation spontaneously occurring in Labrador retrievers eliminates a hydrophobic valine residue (V644del) from the core of the CLZ domain of CNGA3. Both mutations showed perfect segregation with disease phenotype and pedigree and none of the identified alleles were present in 200 control chromosomes from 100 dogs of 18 breeds.

All sequence variants identified during *CNGA3* gene screening were submitted to NCBI dbSNP database (http://www.ncbi.nlm.nih.gov/SNP): both pathogenic variants (c.1931_1933delTGG SS#1067414233 and c.C1270T SS#1067414234) and two newly discovered polymorphisms (c.T1266A silent SS#1067414235 and CFA10:44249374 G>A intronic SS#1067414236).

The *CNGA3* gene product was found to be exclusively expressed in the outer segment of cone photoreceptors in the normal canine retina (**Fig 1C**) and comparative sequence analysis confirmed a high level of sequence conservation among vertebrate genomes for the regions involved (**Fig 1B**). Because the canine protein is 14 aa longer, canine R424 corresponds to human R410 residue, while the canine V644 to V630 of human CNGA3 (**S2 Fig**). For full-length amino acid sequence alignment of human and canine CNGA3 see **S3 Fig**.

### Investigations of CNGA3-R424W mutant channel provide evidence that R424 forms a salt bridge essential for stabilization of channel pore open state

**Fig 2:**
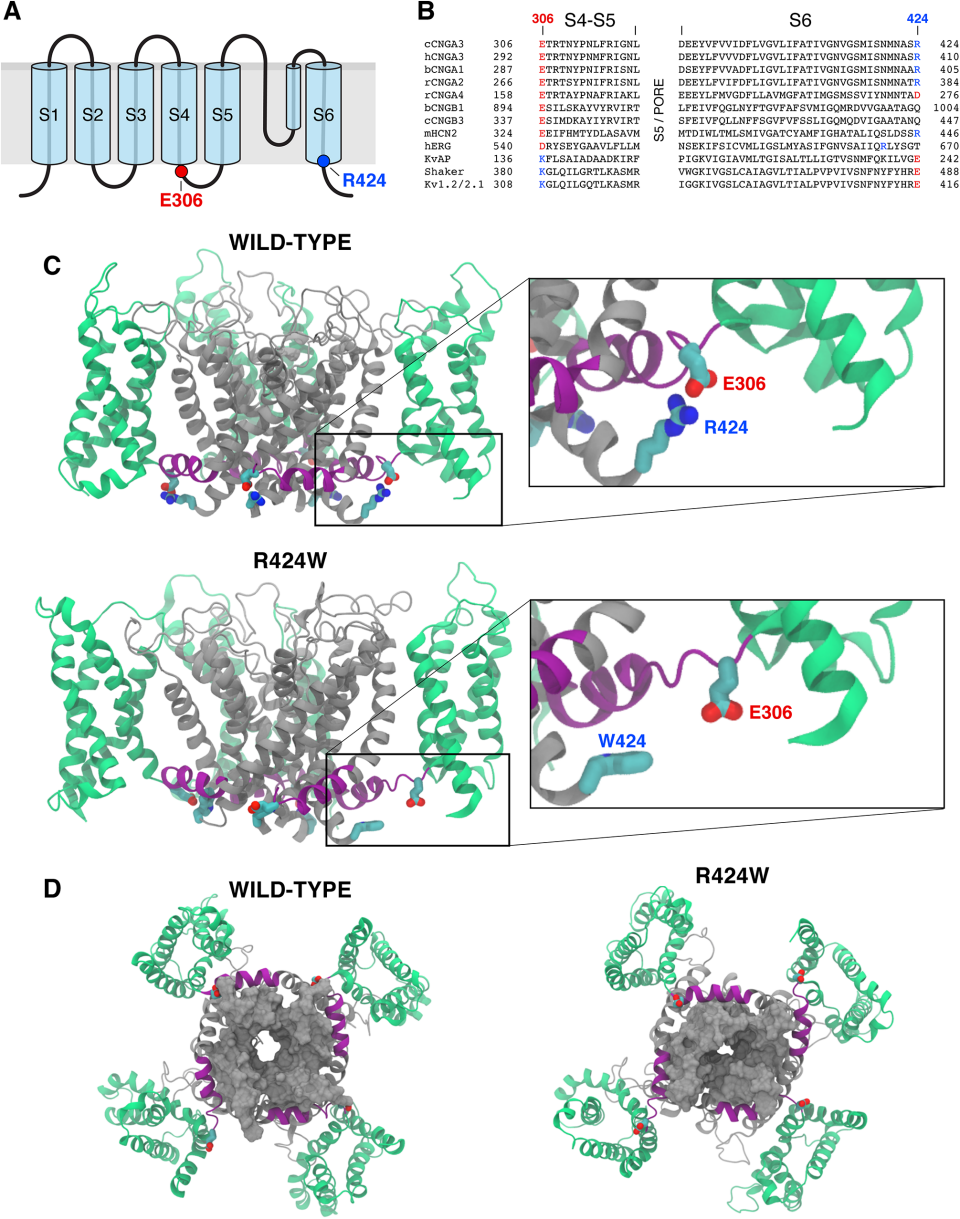
R424W mutation disrupts salt bridge interaction and destabilizes the open state of pore in a homotetrameric CNGA3 model. **(A)** Schematic representation of CNGA3 subunit consisting of six transmembrane (TM) spanning segments (S1-S6) and a pore domain between S5 and S6. The highlighted last residue of S6 (blue) is the site of canine CNGA3-R424W mutation; its predicted partner, glutamic acid E306, is the first residue of S4-S5 linker. **(B)** Amino acid sequence alignment of the S4-S5 linker and S6 segment of selected shaker K^**+**^ channel superfamily members. The TM regions of the CNG channel family were assigned using the crystal structure of the chimeric voltage-gated potassium channel Kv1.2/2.1 (PDB ID: 2R9R). Sequence alignments of S5 domain and pore region were omitted for clarity. The R424 residue is shown in blue and its interacting partner, E306 in red. The conserved salt bridges in the Kv channels show opposite charges at these positions. c = canine, b = bovine, h = human, r = rat, m = mouse. **(C)** Side view of the wild-type CNGA3 homotetramer model and the CNGA3-R424W mutant channel equilibrated in its environment. The voltage-sensing domain (S1-S4) is presented in green, the S4-S5 linker in purple and the pore-forming region (S5-S6) in grey. The residues E306 and R424 are shown as red and blue rods, respectively. The E306:R424 interaction (wild-type) or its loss (R424W mutant) is demonstrated on the higher magnification images. Carbon atoms are labeled in cyan, nitrogens in blue and oxygens in red. Other side chains were omitted for clarity. Note that R424 forms a salt bridge with the E306 molecule in three subunits out of four. **(D)** Bottom views of the wild-type CNGA3 and CNGA3-R424W mutant channels. S6 is represented as a grey solid surface highlighting the partial closure of the pore in the R424W mutant model.

To examine the consequences of the R424W mutation, structural modeling with molecular dynamics and electrophysiological studies were employed. The formerly constructed open state canine CNGA3 (cCNGA3) homology models were used in this study [24]. As described previously, these models were built using the S1-S6 transmembrane (TM) backbone of Kv1.2/2.1 (PBD ID: 2R9R) as a template, because this chimera was identified as the closest protein of known structure to the S1-S6 TM regions of the CNG channels. The putative secondary structure of S1-S6 of cCNGA3 was assigned based on alignment with the voltage-gated K^+^ channel, Kv1.2/2.1 [32] and schematically depicted in **Fig 2A**. This model suggested a salt bridge interaction between R424, the C-terminal residue of S6 and E306, the N-terminal residue of the S4-S5 linker. To investigate the prediction that R424 conserves a salt bridge among CNG channels, the S4-S5 linker region and S6 TM domain of CNG channel subunits were aligned with selected members of the Shaker K^+^ channel superfamily (**Fig 2B**). The residue of interest, R424 is conserved in CNGA1, CNGA2, and CNGA3 subunits as well as in hyperpolarization-activated cyclic nucleotide-gated (HCN) channels. In Kv channels, this site favors an acidic residue (Glu/E). The predicted interacting partner of R424, CNGA3 residue E306, demonstrated a high degree of conservation among the CNG channel subunits and in HCN channels. The Kv channels share the pattern, but with reversal of charges at these positions i.e., the positively charged amino acid residue K in the S4-S5 linker and the negatively charged E at the C-terminus of S6. These observations are consistent with a conserved salt bridge interaction between the S4-S5 linker and S6 in both CNG and Kv channel families.

In order to explore the molecular role of R424 and substantiate the premise of salt bridge formation, the R424 and E306 residues were carefully examined in the CNGA3 homology model relaxed with MD simulations (**Fig 2C and 2D**). First, the MD simulations were used to relax the structure of the wild-type CNGA3 homotetramer channel in its membrane/solution environment for ~130 ns until equilibrated. The simulation was initiated from a complex structure in which all subunits formed the R424-E306 salt bridge. During the entire simulation time, the salt bridge interaction was lost only in one of the subunits, but was maintained intact in the other three subunits (**Fig 2C**). However, with the introduction of the R424W point mutation, all of the salt bridge interactions were lost. Due to the dual hydrophobic/polar nature of Trp (W), these mutant residues did not tend to extend into the solution, but rather stayed at the protein/solution interface, an environment where the hydrophobic moiety could be buried in the hydrophobic core of the protein, but where the polar NH group could stay in a favorable solvated environment (**Fig 2C**). Interestingly, after ~120 ns of simulation, the R424W mutant pore began to close (**Fig 2D**). Full pore closure was not observed over the course of these short simulations, yet our results indicate that the closed state of the channel may be favored in the R424W mutant model (**Fig 2D**).

**Fig 3:**
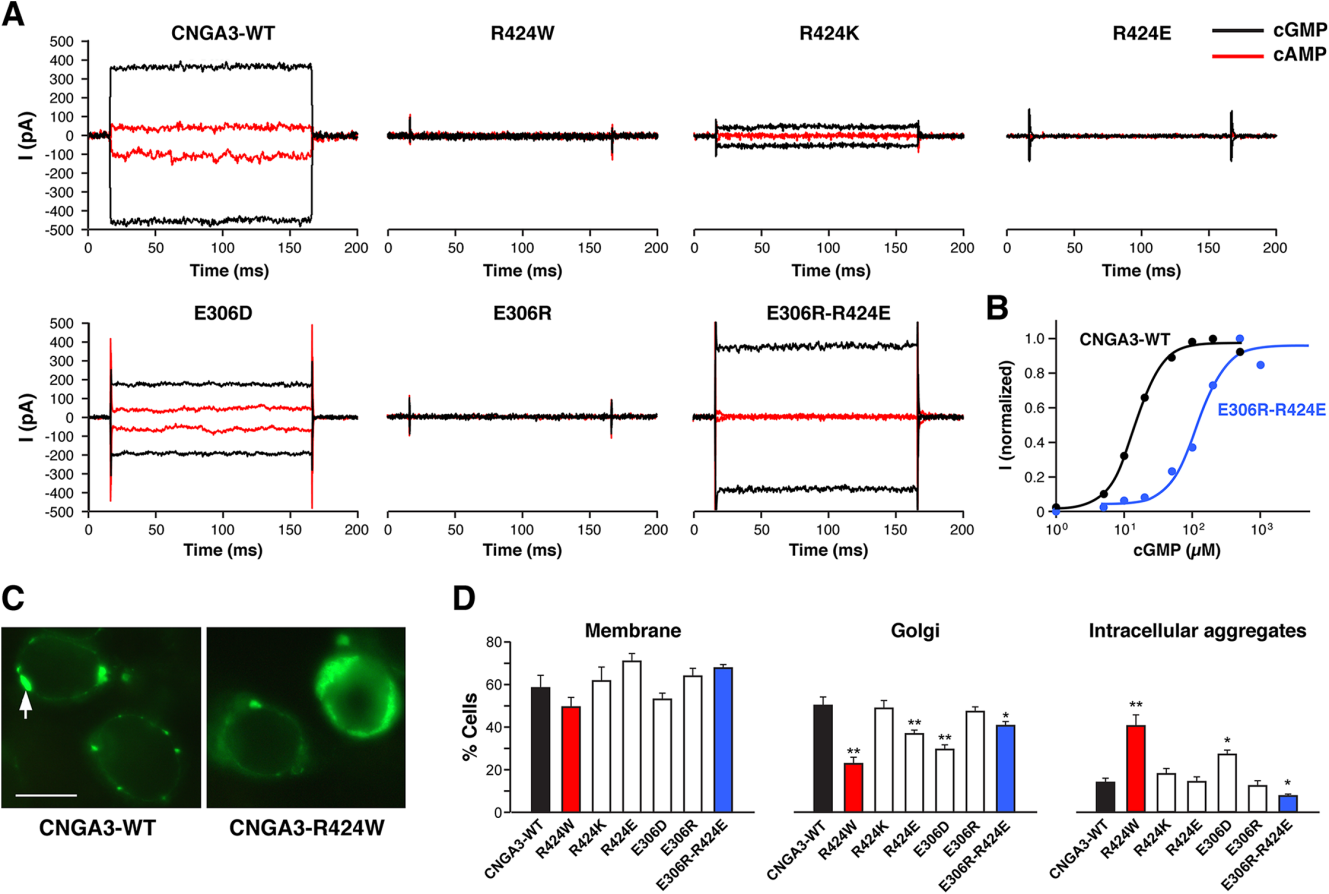
CNGA3-R424W mutant channel is non-functional, but CNGA3-E306R/R424E double charge-reversal mutant rescues the phenotype. **(A)** Cyclic nucleotide-activated currents of the wild-type CNGA3 and a set of R424- and E306-mutant channels recorded from excised membrane patches at -60mV and +60mV in a presence of saturating concentrations of cGMP (200μM) and cAMP (5000μM). No nucleotide-activated currents were recorded for R424W-, R424E- or E306R-mutant channels. E306R-R424E double mutant channel restored cGMP activation, producing large currents similar to the wild-type CNGA3. Detailed results from multiple patches are summarized in S2 Table. **(B)** cGMP dose-response relationship of the wild-type and CNGA3-E306R-R424E double charge-reversal mutant channels. The plot shows the ligand concentration-dependent activation of the wild-type (black) and CNGA3 double mutant (blue) homomeric channels at -60 mV. Each data set was taken from a representative single patch. The cGMP K_0.5_ is 14.2 μM for the wild-type and 118.4 μM for the mutant channel; Hill coefficient [N_h_] value is 2.1 and 2.0, respectively. **(C)** Cellular localization of YFP-tagged wild-type canine CNGA3 and CNGA3-R424W mutant in HEK tsA201 cells. Cells transfected with the wild-type construct showed specific fluorescent signals in the plasma membrane and Golgi-like organelles (arrow); cells expressing the mutant protein exhibited augmented intracellular signals consistent with aggregate formation in addition to membrane and Golgi-like fluorescence. Scale bar: 10μm. **(D)** Histograms of averaged cellular localization patterns for R424- and E306-mutant constructs *versus* wild-type CNGA3. A significant increase in intracellular aggregates was found in R424W-transfected cells (red bars) *vs* CNGA3-WT (black bars), and an apparent reduction in aggregate formation in the E306R-R424E double mutant channels (blue bars). An unpaired t-test was used to compare individual mutants *vs* WT (mean% ± SD for n>300 cells). * p-value of <0.05; ** p-values <0.0001.

To gain more insights into the CNGA3-R424W mutant channel function, a series of R424- and E306-targeted site-directed mutant constructs were examined and assessed using patch clamp technique as previously described [23, 24]. Based on the results from MD trial suggesting a crucial role of R424-E306 salt bridge in the stabilization of the open state of the channel, it was expected that R424- and E306-targeted mutations would either result in abolished channel function or its preservation depending on the side-chain charge design. Supporting salt bridge hypothesis, the canine ACHM2-associated mutation R424W as well as the charge reversal mutations, R424E and E306R, produced no cGMP- or cAMP-activated currents (**Fig 3A**). On the other hand, R424K and E306D, the side-chain charge-conserving mutant constructs, were thought to have the potential to preserve channel function albeit with possible alterations in the channel properties. Indeed, the E306D and R424K mutant channels resulted in either cGMP- and cAMP-activated currents with cAMP/cGMP efficacy values similar to those of CNGA3-WT, or diminished cGMP-activated currents with no cAMP-activated currents, respectively (**Fig 3A**). Finally, the double charge-reversal mutant channel, E306R-R424E, was assessed for a potential rescue of the channel function. As expected, the E306R-R424E restored cGMP activation producing large currents similar to the CNGA3-WT channel; however this double mutant was unresponsive to the partial agonist cAMP with an efficacy in the WT channels of ~20% compared to cGMP activation (**Fig 3A**). Dose-response relations of cGMP-activated currents for the CNGA3-WT and E306R-R424E double mutant channels are shown in **Fig 3B** and the average K_0.5_ values are summarized in **S2 Table**. The dose-response curves for cGMP activation showed an average K_0.5_ value of ~102 μM for E306R-R424E mutant, which is ~8 fold higher than that of the CNGA3-WT, 12.3 μM (**S2 Table**). The loss of cAMP-activated currents in all R424-mutant channels examined and the cGMP K_0.5_ shift in the E306R-R424E double charge-reversal mutant channel, suggest that changes in the S4-S5 linker to S6 salt bridge alter nucleotide recognition and gating. However, since the K_0.5_ values reflect both nucleotide binding and pore opening, deeper insights into the properties of this double mutant will need to be addressed in future studies.

Since documented ion channelopathies are often associated with misfolding and/or mistrafficking of the affected membrane proteins, the subcellular localization patterns of YFP-tagged R424- and E306-mutant channels were studied *in vitro*. Representative photomicrographs of CNGA3-WT and CNGA3-R424W channels are shown in **Fig 3C**. The wild-type channels showed specific expression and trafficking to the plasma membrane and Golgi-like organelles with few cytoplasmic aggregates. In contrast, cells transfected with CNGA3-R424W demonstrated a significant increase in intracellular aggregates and a reduction of Golgi-like expression suggesting ER retention and altered protein folding (**Fig 3C**). The averaged subcellular fluorescence data for all R424- and E306-mutant channels are summarized in **Fig 3D**. No significant variation in abundance of membrane expression was observed among the mutant constructs; however, cells transfected with R424W, R424E and E306D revealed reduction of Golgi-specific expression, while the highest percentage of cells with intracellular aggregates was calculated for R424W and E306D mutant channels. Notably, the double charge-reversal mutant, CNGA3-E306R-R424E, exhibited a fluorescence pattern similar to CNGA3-WT with a significant reduction in intracellular aggregates (**Fig 3D**).

### The V644 deletion alters CLZ core structure and results in unfolding of coiled-coil complex

**Fig 4:**
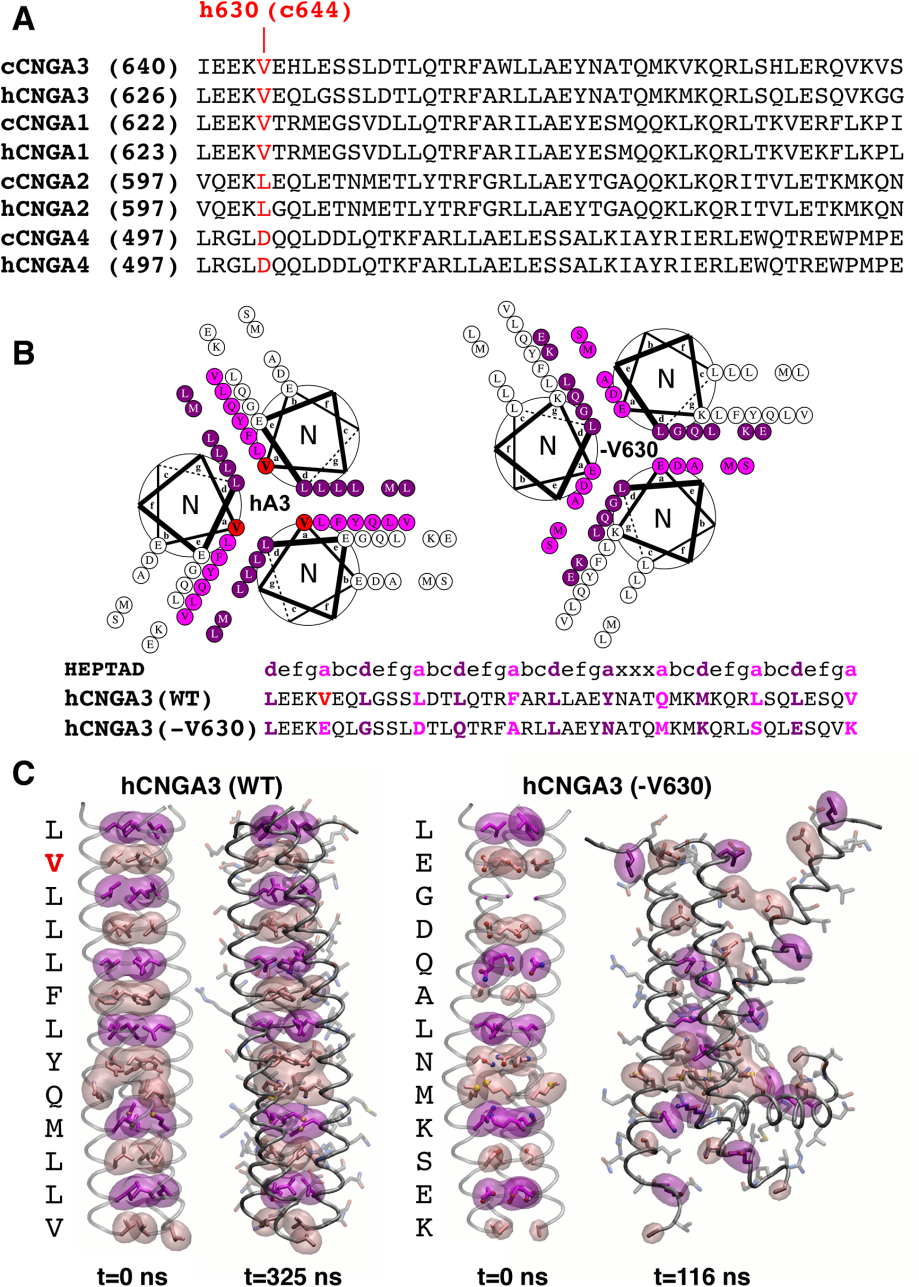
The V644 deletion alters CLZ core structure indicating unfolding of coiled-coil complex. **(A)** Sequence alignment of the CLZ (C-terminal leucine zipper) domains in CNGA-type subunits and conservation of the V644 residue. Note that canine V644 corresponds to V630 of human CNGA3. c = canine; h = human. **(B)** Helical wheel diagrams looking down the superhelical axis of the CLZ coiled-coil from the N- to C- terminus. Heptad *a* positions are marked in pink, *d* positions are denoted in magenta, and V630 is shown in red. The V630del shifts residues from *b* and *e* heptad positions to the core *a* and *d* positions and causes the predominantly hydrophobic residues of the coiled-coil core in the wild-type structure (left) to shift destabilizing charged and small residues in the mutant complex (right). Sequence diagram of the wild-type and V630del mutant heptad is shown below. **(C)** Molecular dynamics trajectories and models of the wild-type and mutant CLZ trimeric structures. Snapshots before (t = 0 ns) and after (t = 325 ns) simulation for the wild-type CNGA3-CLZ (left). The CLZ core clearly remains intact with small perturbations near the solvent exposed N- and C- termini over long timescales. Snapshots before (t = 0 ns) and after (t = 116 ns) simulation for the V630del mutant model (right). The V630 deletion dramatically alters the previously stabilizing residue-residue interactions, leading to disruption of the CLZ coiled-coil and helical structure. Small red and blue spheres correspond to oxygen and nitrogen atoms, respectively. See also **S4**and **S5 Figs** for the full-length molecular dynamics simulations.

The second novel canine disease model of ACHM2 is causally associated with Val644 deletion, a residue located in a coiled-coil domain of the CLZ in CNGA3. This 47-amino acid CLZ sequence is found in all CNG A-type subunits, but not in the CNGB1 or CNGB3. CLZ mediates subunit interactions among the A-type subunits through a trimeric coiled-coil structure during channel assembly in the 3A3:1B3 stoichiometry in the cone outer segment membranes [3, 4]. The spontaneous mutation identified in the Labrador retriever breed affects an essential hydrophobic V644 residue that is conserved in CNGA1 and CNGA3 subunits between human and canine orthologs (**Fig 4A**).

A high-resolution X-ray crystal structure (PDB ID: 3SWY) of the human CNGA3 (hCNGA3) CLZ domain (sequence identity with canine CNGA3 CLZ: 83%) provided a template for exploring the V644del in the canine CLZ using MD simulations. As depicted in **Fig 4B**, human CNGA3-V630 residue (equivalent to canine V644) is in an *a* heptad position in the second turn of the coiled-coil domain. The trimeric CLZ domain coiled-coil structures of CNGA3-WT and CNGA3-V630del were constructed using the relaxed backbone of the hCNGA3-CLZ domain and their stability was assessed qualitatively by MD simulations. The wild-type CLZ structure appeared structurally stable, at least on the 325 ns timescale examined here (**Fig 4C**). The WT coiled-coil core remained intact and tightly compact with the exception of fluctuations of the last *a*-position valine, likely due to solvent exposure and typical end-effects. The V630del mutant, however, exhibited rapid loss of the coiled-coil and tertiary structure over the course of only 100 ns, with unfolding beginning at the C-terminus within 30 ns, followed by the N-terminus within 50 ns. From the model structures alone, it was clear that the CLZ-V630 deletion mutant could significantly destabilize the coiled-coil assembly, unfolding the helical turns near the mutation at best and potentially leading to complete unfolding of the CLZ domain at worst. The average backbone root-mean-square deviation (RMSD) calculated over the entire trajectory was 9.06Å, with a maximum of 12Å when compared to the deletion model’s initial backbone configuration, a clear sign of structural instability (**Fig 4C**), further highlighted by the wild- type’s 1.54Å average backbone RMSD over a similar simulation timescale. For the full-length video recordings of MD trajectories of hCNGA3-WT and V630del mutant CLZ trimeric structures see **S4**and **S5 Figs**.

**Fig 5:**
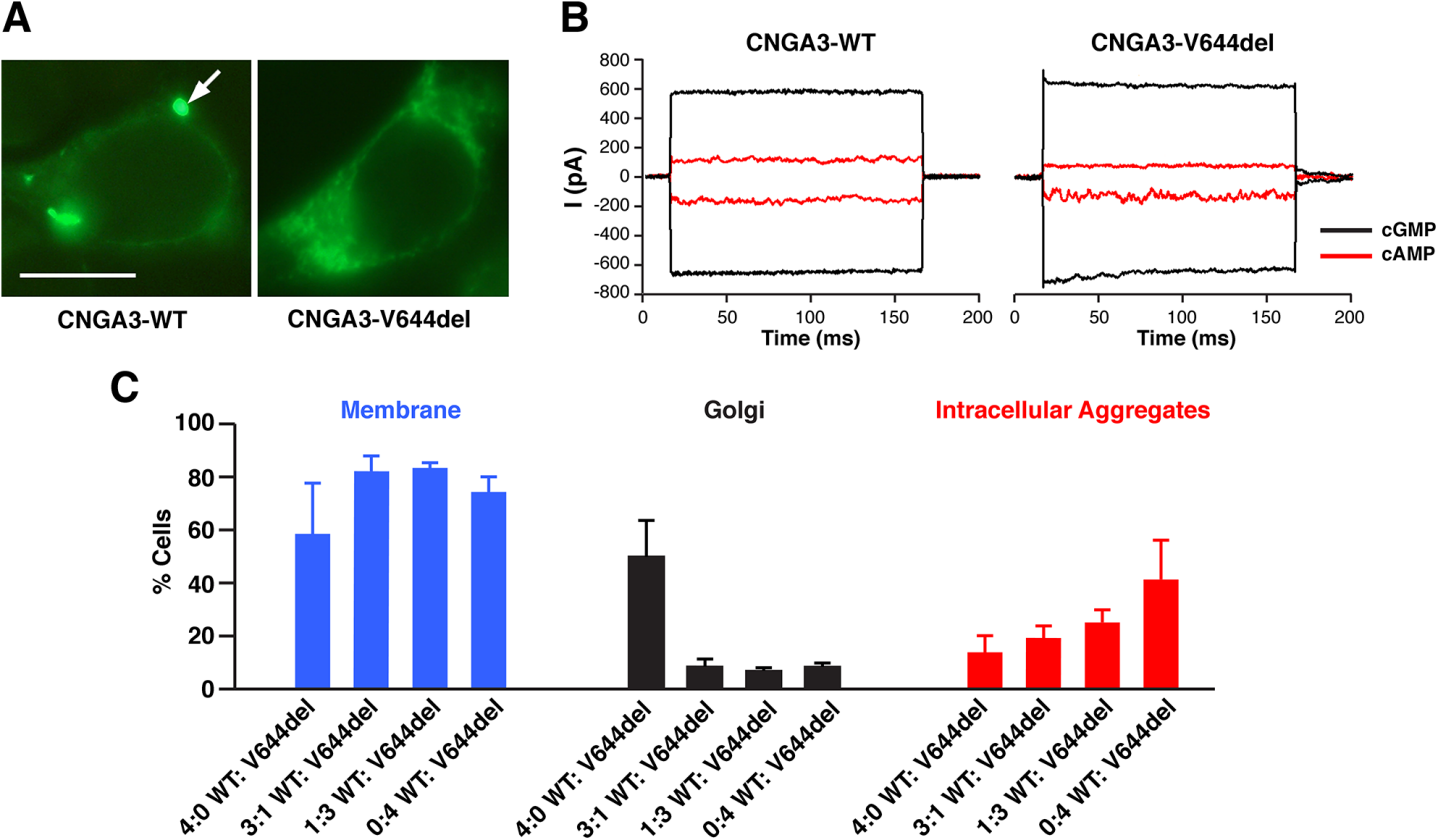
Complex cellular phenotype of V644del mutant channel. **(A)** Cellular localization of YFP-tagged wild-type canine CNGA3 and CNGA3-V644del mutant in HEK tsA201 cells. Cells transfected with the wild-type construct showed specific fluorescence pattern of expression limited to the plasma membrane and Golgi-like organelles (arrow); an evident increase in intracellular aggregates was observed in cells transfected with V644del mutant construct consistent with abnormal trafficking and potential ER retention. Scale bar: 10μm. **(B)** cGMP- and cAMP-activated currents recorded from CNGA3-WT and a responsive patch expressing V644del mutant channels. Approximately 40% of V644del mutant patches had no cGMP-activated currents, in contrast to 100% responsive patches from the CNGA3-WT-transfected cells. This partial loss of channel activity might reflect incomplete subunit assembly associated with disruption of the coiled-coil structure as depicted in the simulation studies. The responsive patches showed cyclic nucleotide-activated currents with similar characteristics to WT channels. **(C)** Histograms of subcellular localization patterns monitored in HEK tsA201 cells co-transfected with V644del and CNGA3-WT cDNA constructs. Cells were transfected with either CNGA3-WT or CNGA3-V644del or both constructs at the indicated ratios. Each cell count represents >300 cells from at least 2 transfections (mean% ± SD).

In addition to MD simulations, the effects of V644del were examined with regards to its subcellular localization and electrophysiological properties. In the previous studies on CNGA3 S1-domain mutant models an increase of intracellular aggregates was observed in all ACHM2-associated mutant channels examined and this predominant fluorescence distribution colocalized with ER-specific marker, GRP94 [23]. Similarly, the fluorescence pattern of V644del showed an increase in the intracellular aggregates in comparison to the CNGA3-WT suggesting an abnormal membrane protein trafficking possibly related to improper folding or subunit assembly (**Fig 5A**). A series of patch clamp recordings of CNGA3-V644del channels revealed that approximately 40% of the mutant patches were unresponsive with no cGMP-activated currents, in contrast to 0% unresponsive patches from the CNGA3-WT-transfected cells. This reduction in the number of responsive patches might indicate a failure of proper subunit assembly due to disruption of the coiled-coil structure as illustrated in the simulation studies. However, 60% of patches showed cyclic nucleotide-activated currents, and these responsive mutant patches exhibited currents indistinguishable from the wild-type channels (**Fig 5B**). The mean cGMP-activated currents for CNGA3-WT were -375pA (SEM ± 78pA; n>10), while those for CNGA3-V644del were -417pA (SEM ± 83pA; n>10) with the cAMP efficacy of ~20% for both CNGA3-WT and the responsive subgroup of V644del mutant channels.

To explore the potential effects of CNGA3-V644del on the wild-type subunit folding and assembly, CNGA3-WT and CNGA3-V644del mutant constructs were co-transfected at varying ratios. The histogram in **Fig 5C** demonstrates that increasing the V644del component in the WT:V644del mixture resulted in a dramatic reduction of fluorescence associated with Golgi-like structures and a gradual increase in intracellular aggregates. However, regardless of the WT:V644del ratio, the plasma membrane fluorescence was comparable in all cells examined. We questioned whether cells with cGMP-activated currents might retain membrane fluorescence by expressing “rogue” subunits improperly assembled into a channel. This assumption would be consistent with the 40% of V644del mutant patches exhibiting no channel function.

**Fig 6:**
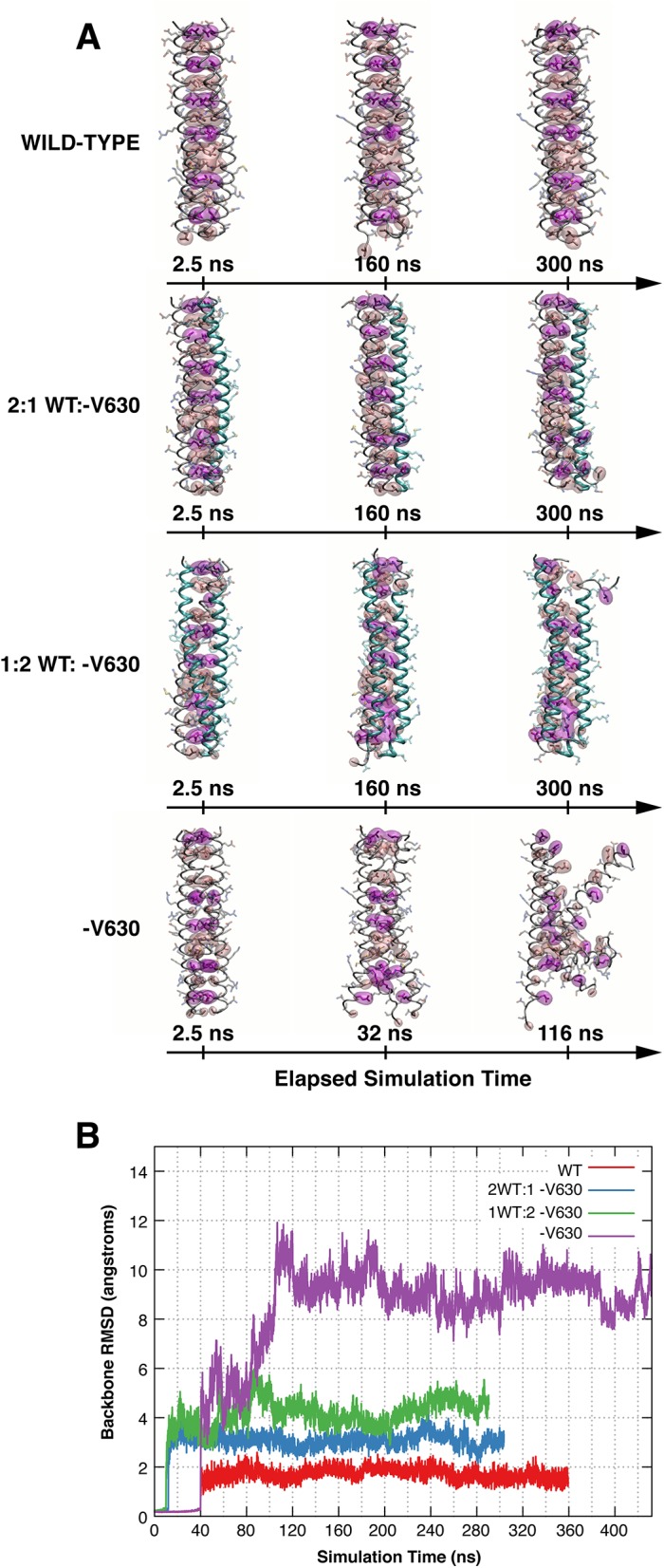
Long timescale molecular dynamics simulations of the native CLZ, V630del mutant structure, and 2:1 and 1:2 WT to V630 hybrid deletion models. **(A)** Molecular dynamics trajectories of the wild-type, V630del mutant and V630 hybrid CLZ deletion models at the selected time points. The wild-type CLZ structure exhibited long timescale stability. The core structure of the coiled-coil remained intact during the entire trajectory, leading to an impressive long timescale stability and average backbone root-mean-squared deviation (RMSD) of 1.54 Å. The 2:1 WT:V630del CLZ structure showed some unfolding. The core structure of the coiled-coil retained two thirds of its wild-type structure, leading to a greater overall stability compared to the all V630del structure. However, the model did exhibit some unfolding with the V630del helix (cyan) beginning to detach from the complex. The 1:2 WT:V630del CLZ complex showed more unfolding. The core structure of the coiled-coil contains charged amino acids at opposing helical positions: 630E_a_ 637D_a_ 658K_d_ 665E_d_ 669K_a_ causing significant disruption at these positions. The V630del CLZ structure unfolded quickly. The core structure of the coiled-coil, now composed of destabilizing small and charged amino acids, begun to unfold shortly after initialization of the simulation. After 100 ns, most of the tertiary structure was lost, leaving an amalgam of helices and an average backbone RMSD of 9.09Å. Space filling surfaces: core *a* (pale pink) and *d* (magenta) positions applies to all panels. **(B)** Root-mean-squared deviations of the backbone structure at time t = x ns compared to the starting structure. The wild-type structure exhibits impressive stability with an average RMSD of 1.54 Å; the hybrid structures have intermediate stability with average RMSDs of 2.99 Å and 4.04 Å for the 2:1 (blue) and 1:2 (green) models, respectively. The 3-fold V630 deletion model is clearly unstable (purple) having an average backbone RMSD of 9.06 Å.

To further examine structural stability of CNGA3-WT:V644del at different stoichiometric ratios, intermediate CLZ trimer models were evaluated using fully atomistic classical MD simulations. The stability of two hybrid structures composed of helical subunits from the hCNGA3-WT and V630del was qualitatively assessed in comparison to the established wild-type structure and the inherently unstable mutant model (**Fig 6**). Both structures exhibited enhanced stability when compared to their all-deletion counterparts but also did show some signs of unfolding during the evolution of the trajectory (**Fig 6A**). Predictably, the 2:1 (WT:V630del) structure, which retained two thirds of the hydrophobic core packing and avoided the electrostatic repulsion introduced in the all-deletion mutant, was more stable than its 1:2 (WT:V630del) counterpart with an average backbone RMSD of 2.99 Å versus 4.05 Å (**Fig 6B**).

## Discussion

We performed a phenotype-directed candidate gene analysis to determine the genetic basis and explore the molecular mechanism of congenital achromatopsia found in the German shepherd and Labrador retriever breeds. Our studies led to the discovery of two novel canine mutations in *CNGA3* encoding a subunit essential for the generation of light-evoked electrical responses in cone photoreceptor outer segment membranes through CNG channels. The R424W and V644del mutations in *CNGA3* represent the first canine models of ACHM2-associated channelopathies. To identify potential factors contributing to the pathological mechanism of the disease, we used a combination of experimental and structural modeling approaches coupled with *in vivo* findings. Here, we discuss the role of the R424 residue in stabilizing the channel open state through a conserved salt bridge interaction with E306, followed by pathophysiological consequences of V644 deletion and its impact on the trimeric coiled-coil interactions and channel assembly.

### Direct evidence of R424-E306 salt bridge formation and its role in stabilization of the open pore in CNG channel

Previous biophysical, biochemical and structural studies have provided detailed molecular insights into the electromechanical driving forces involved in Shaker-like channel voltage-dependent gating [45, 46]. The voltage-sensing domain (VSD) of Shaker channels is comprised of S1-S4 domains and the S4 helix bears a large number of positive charges. The S4 helix responds to changes in external voltage by vertical displacement [47]. Upon membrane depolarization, the S4 moves outward, dragging the interfacial S4-S5 linker with the S6 helix, leading to pore opening; whereas upon hyperpolarization, S4 moves downward pushing on the S4-S5 linker and directing S6 to close the pore.

The first evidence for a direct interaction between the S4-S5 linker and distal residues in S6 was reported by Lu and coworkers [48]. Since their seminal experiments, other research groups confirmed this initial work and the current consensus proposes that the residues forming the S4-S5 linker and the distal region of S6 are finely tuned to permit a close interaction [46]. According to these studies, the tight coupling between voltage sensor activation and pore opening is lost upon introducing mutations in either region, yet function can be restored when introducing compensatory mutations in both regions. In hERG channels, an interaction between the S4-S5 linker and S6 was proposed to stabilize the VSD/pore coupling [49]. Interestingly, the positions in hERG do not correspond exactly to those in CNGA3. The equivalent residue to E306 in CNGA3 is a negatively charged Asp (D540) in hERG and its proposed counterpart in S6 is an Arg (R665) five positions above the CNGA3-R424 residue. More recent work suggested the involvement of hydrophobic interactions in the electromechanical coupling between the S4-S5 linker and the S6 of these channels [50].

The gating of CNG channels has remained somewhat of a mystery. Indeed, this channel family has charged residues in the VSD; however, the gating is voltage-independent but driven by the binding of cyclic nucleotides, each subunit possessing a nucleotide-binding domain (NBD). Based on our previous work suggesting involvement of the S4-S5 loop and S6 in voltage-activated gating [24], we examined CNGA3 in comparison to the voltage-gated like (VGL) superfamily. It was immediately apparent that R424 aligned at the C-terminal region of S6, the helix that forms the ion permeation pore. Furthermore, as illustrated by multiple sequence alignment, the acidic residue E306 in CNGA3 located at the N-terminus of the linker and might interact directly with R424 (Fig 2B). This pattern was strictly conserved in CNGA1, CNGA2 and in HCN subunits and we hypothesized that R424 might be involved in a salt bridge interaction crucial for folding, subunit assembly and channel gating. Indeed, the spontaneous R424W mutation occurring in the ACHM2 dogs described here disrupted the salt bridge interaction when examined in the *in vitro* system, and resulted in destabilization of the open state of the pore in MD simulations. Direct experimental support for the salt bridge formation was obtained from the R424- and E306-targeted mutants study in which reversal of either the S4-S5 linker or the S6 side-chain charge resulted in a loss of channel function with restoration of channel activity in the double charge-reversal mutant E306R-R424E.

Additional insights regarding the molecular defects of the R424W mutation were derived from cellular localization studies. Although cells transfected with R424W mutant construct showed an increase in intracellular aggregates (from ~10% in the wild-type to ~40% in the R424W mutant), the expression profile of plasma membrane fluorescence did not vary significantly. These results differ from a previous canine ACHM missense mutation, CNGB3-D262N, located in the S2 transmembrane segment of CNGB3 [24, 51]. In these studies, 100% of cells expressing the D262N mutation showed intracellular aggregates with a very few (<10%) cells expressing membrane or Golgi-like fluorescence. Thus, although protein misfolding and mistrafficking contribute to the CNGA3-R424W phenotype, our results suggest that the loss of an essential interaction between the S4-S5 linker and S6 might be the leading factor of channel malfunction observed in CNGA3-R424W mutant, manifesting in a loss of cone photoreceptor function in CNGA3-R424W-affected dogs.

### Gating mechanism of CNG channels

In CNG channels, the S2, S3 and S4 TM segments bear a large number of positive and negative charges. In contrast to the Shaker channels, the positive charges are not confined to the S4 segment, but are dispersed throughout the VSD sequence. Recently, we proposed that the charges of both signs form an electrostatic zipper that in effect laces the charge interactions locking the VSD of CNG channels into an immobile state [24]. The present study demonstrated that channel activity was abolished with the loss of an acidic residue at CNGA3-E306 or a basic residue at CNGA3-R424, yet swapping the charges in the E306R-R424E double mutant restored channel function albeit with altered nucleotide recognition. Therefore, if the VSD is locked in its activated state and if there is a strong coupling between the S4-S5 linker and the distal region of S6, we propose that the pore is continually stabilized in its open state.

Although the exact mechanism of CNG channel gating involving the intracellular nucleotide-binding domain (NBD) remains to be determined, structural studies revealed that the NBDs form a tetrameric ring below the transmembrane pore that interacts via the C-linker with the S4-S5 linker [52–55]. Structural studies of a prokaryotic nucleotide-activated channel, MlotiK1, demonstrated that upon cAMP binding, the NBD moves toward the membrane [56] while the pore helices remain in an open configuration in both the ligand-bound and unbound states. In cone CNG channels, cAMP-activated currents are smaller than cGMP-activated currents with a much higher K_0.5_ suggesting coupling differences between the ligand binding and channel gating between these two ligands. The nucleotide activation properties of the E306R-R424E double mutant provides clear evidence for differences in the communication of ligand binding information to regions of the channel involved in gating, possibly with the S4-S5 loop and S6 salt bridge. Since cAMP and cGMP differ only at the C2 and C6 positions on the purine, the double mutant current profiles eventually might contribute to a deeper understanding of how ligand recognition and contacts within the binding domain are coupled to conductance properties.

### The CNGA3 V644 deletion alters CLZ core structure and impairs the stability of coiled-coil complex

Coiled-coil structures, including the CLZ domains, are one of the most widely observed helical motifs among proteins [57, 58]. The sequences of coiled-coils are characterized by seven-residue ‘heptad’ repeats [abcdefg]_n_ caused by winding the canonical alpha-helix from 3.6 to 3.5 residues per turn. Every seventh residue then lies on the same face of the coil’s helices, giving rise to the familiar periodicity from which the coil derives its structure and stability [59]. In such structures, the core *a* and *d* positions (positions that point toward neighboring helices or into the center of the helical assembly) are tantamount to the coiled-coil. The core residues are often characterized by their tight “knob-into-holes” or “zipper like” packing making van der Waals contacts with neighboring core side-chains, and often encode both the orientation and oligomerization state of the folded structure [58, 60]. Typically, in water-soluble coiled-coils, the core positions are highly hydrophobic, as is the case in the CLZ domain, which is composed primarily of the branched amino acids valine and leucine a well known combination leading to parallel-trimeric configurations. Thus, it is expected that even a slight interruption or modification of the core repeat can dramatically alter the structure of the CLZ.

We used a robust statistical protein design algorithm to identify the most energetically favorable configurations of the side-chain rotamers for both the wild-type CNGA3 and V644del mutant models [41, 42, 61]. The MD simulations using the hCNGA3 deletion mutant model demonstrated that the cCNGA3-V644del mutation could significantly destabilize the coiled-coil assembly of CLZ domains. As opposed to a core mutation in which the heptad repeat must accommodate a few changes in residue-residue interactions, a deletion has a much more profound effect on the structure. All residues following the deletion must “shift” one heptad position, disrupting nearly all residue-residue interactions that stabilize the wild type structure. The polar and charged amino acids typically found at the *b* and *e* heptad positions now populate the core of the coiled-coil and exhibit significant electrostatic repulsion. Larger residues are unable to pack in the core without expansion of the coil’s radius, while “small” residues (G, A and S), do not provide the packing and van der Waals contact necessary to stabilize the core. Therefore, we hypothesize that the CNGA3-V644del mutant subunits *in vivo* do not form proper CNGA3 subunit assemblies essential for stable channel biogenesis and, as discussed below, an altered N- to C-terminal interaction may also contribute to the ACHM phenotype segregating in the Labrador retriever breed.

It should be noted, however, that the MD simulations were not performed to examine how individual CLZ domains with the deletion would assemble or behave. Instead, we initiated the simulations with the assembled coiled-coil structure of mutant CLZ domains, which led to disruption of the assembly. Moreover, the simulations were performed only with the CLZ domain structure, missing the VSD, pore-forming region, C-linker, and cyclic NBD. Thus, it is still to be determined how misassembly of the CLZ domain coiled-coil structure caused by this mutation would affect the whole channel structure and function. Nonetheless, the simulations suggest that significant interruption of the N-terminal region of the CLZ domain is a direct result of the V644del mutation.

### The newly identified canine CNGA3 mutant models provide important insights into human ACHM2-associated channelopathies and their treatment

Spontaneously occurring animal models of inherited retinal diseases are vital for studying the nature of these molecular defects, and pivotal for assessment of experimental therapies [15, 31, 62–64]. rAAV-mediated treatments for human retinal degenerative disorders developed and tested in natural canine models have already been successfully translated and have entered the final phases of clinical trials [65–68], or are in the final stages of pre-clinical development [63]. The existing animal models for CNG-associated ACHM have greatly contributed to the development of treatment and the initial proof-of-concept studies [15, 17, 18, 21, 22, 69]. However, except for cpfl5 mouse [70], the available *CNGA3* models represent either null phenotype (CNGA3^**-/-**^ mouse model) [71] or premature stop (R236X sheep model) [22, 72], and are obvious targets for AAV-mediated gene augmentation therapy. The novel canine models of ACHM2, however, carry mutations that cluster in the C-terminal mutational hotspot of the molecule, a region critical for channel gating and tetramerization. As such, they represent the majority of human *CNGA3* mutation classes (S2 Fig and S3 Table) and thus constitute an important model system to conduct such treatment validation studies. Both the *in vitro* and *in silico* analyses of the novel canine *CNGA3* mutations indicate that introduction of the wild-type gene results in phenotype rescue, either through reduction of intracellular aggregates or by increasing the stability of CLZ complexes. However, this premise needs to be tested experimentally in the live animal through vector mediated gene augmentation.

An R410W mutation analogous to the canine R424W was found to be causatively associated with loss of cone function in human ACHM2 [25]. The authors reported an R410W/V529M compound heterozygous patient with absent cone ERG responses, severely reduced visual acuity, photoaversion and nystagmus. Both R410W and V529M mutations contributing to this phenotype eliminate cGMP-activated currents while examined *in vitro* [73]. More recently, an unrelated ACHM2 patient of European descent was reported; this patient harbors a homozygous R410W mutation identical to the canine R424W model, and exhibits a complete loss of cone function similar to the CNGA3-R424W-affected dogs [26].

A pathogenic hCNGA3 missense mutation affecting a conserved hydrophobic residue within the CLZ domain was described by Goto-Omoto and colleagues [74]. The reported patient, diagnosed with complete achromatopsia, harbored a R436W/L633P compound heterozygous mutation, the latter located in a *d* heptad position, only one turn downstream from V630. Using an experimental *Xenopus laevis* oocyte model, Dai and Varnum [27] demonstrated that CNGA3-L633P mutant subunits, either alone or co-expressed with CNGB3, showed little change in cGMP activation but exhibited large changes in the regulation of cGMP sensitivity in the presence of phosphoinositides (PIPs), which are known to affect coupling between the amino- and carboxy-termini of CNGA3 subunits modulating physiological feedback in cones [75]. Consistent with the results presented here, the L633P mutation reduced, but did not eliminate expression of functional CNG channels *in vitro*. Although not investigated here, future studies on the co-expression of the CNGA3-V644del and CNGB3 subunit will aim to determine if aberrant channel regulation and changes in the PIP feedback, similar to those seen with the human CNGA3-L633P mutation, contribute to the loss of cone function observed in V644del-affected dogs in addition to the structural consequences of this mutation presented here.

Taken together, our *in vitro* findings indicate that the CLZ domain is involved in long-range intersubunit interactions important for channel regulation in the cone outer segments, and the V644del described here, similar to the L633P reported in patients, may produce a complex change in channel activation properties. More importantly, such cryptic regulatory features are not readily revealed while examined in the *in vitro* system. *In vivo* studies utilizing the canine CNGA3-R424W and-V644del models for translational purposes are currently under development.

## Supporting Information

S1 Fig Clinical evidence of achromatopsia in R424W mutant based on behavioral vision testing under photopic and scotopic light conditions. Subjective vision testing shows complete loss of vision during daylight hours, but normal visual performance under dark, twilight, artificial, and low-level fluorescent light conditions. Movie illustrates visual behavior in the R424W-affected dog that is similar to what was observed in dogs homozygous for V644del mutations.
(MOV)

S2 Fig The Proposed CNGA3 Protein Model Places Two Novel Canine ACHM2 Mutations in the C-Terminal Mutational Hotspot. Schematic representation of human CNGA3 molecule deduced from multiple sequence alignments. Canine R424 residue affected in German shepherd with achromatopsia is located at the terminal cytoplasmic end of S6 (arrow) and corresponds to R410 of the human CNGA3. The V644 mutated in Labrador retriever is positioned within CLZ domain and corresponds to hCNGA3-V630 (arrow). All known human disease-associated mutations are denoted and color-coded. Mutations in homo-, hetero- as well as both, homo- and heterozygous states are scattered throughout all domains with mutational hotspot located in the C-terminal part of the protein. See also **S3 Table**) for additional details.
(TIFF)

S3 Fig Amino acid sequence alignment between human (h) and canine (c) CNGA3. Orthologs of human (NCBI# NP_001289.1) and canine (NCBI# NP_001288041.1) CNGA3 demonstrate >82% of amino acid sequence identity with the highest degree of conservation in the C-terminal half, a region affected by the two novel canine mutations (arrows). Evolutionarily conserved residues are highlighted in red. h = human, c = canine; Sequence alignment was prepared using *Mutlalin* v.5.4.1.
(TIFF)

S4 Fig Video recording of the molecular dynamics trajectory of the hCNGA3-WT CLZ trimeric structure. All simulations were run with a temperature of 310 K fixed using Langevin dynamics and a pressure of 1.0 atmosphere using the Nose-Hoover Langevin piston pressure control. The wild-type CLZ structure appeared stable during the entire 325 ns trajectory leading to impressive long timescale stability and an average backbone root-mean-squared deviation (RMSD) of 1.54 Å. The WT coiled-coil core remained intact and tightly compact with the exception of fluctuations of the last *a*-position [33], likely due to solvent exposure and typical end-effects that are not unexpected.
(MOV)

S5 Fig Video recording of the molecular dynamics trajectory of the hCNGA3-V630del mutant CLZ trimeric structure. All simulations were run with a temperature of 310 K fixed using Langevin dynamics and a pressure of 1.0 atmosphere using the Nose-Hoover Langevin piston pressure control. The V630del mutant exhibited rapid loss of the coiled-coil tertiary structure over the course of about 100 ns, with unfolding beginning at the C-terminus within 30 ns, followed by the N-terminus within 50 ns. After 100 ns, the tertiary structure is mostly lost, leaving an amalgam of helices and an average backbone RMSD of 9.09Å.
(MOV)

S1 Table List of primers and PCR conditions used for canine *CNGA3* gene analysis. (DOCX)

S2 Table Summary of cyclic nucleotide-activated currents recorded for CNGA3-WT and a set of R424- and E306-mutant channels. (DOCX)

S3 Table A comprehensive summary of human *CNGA3* variants and their phenotypic consequences. (DOCX)
