# Supplementary figures and images for: Canine CNGA3 Gene Mutations Provide Novel Insights into Human Achromatopsia-Associated Channelopathies and Treatment
Source: PLoS One. 2015 Sep 25;10(9):e0138943. doi: 10.1371/journal.pone.0138943 (PMC4583268; doi:10.1371/journal.pone.0138943)

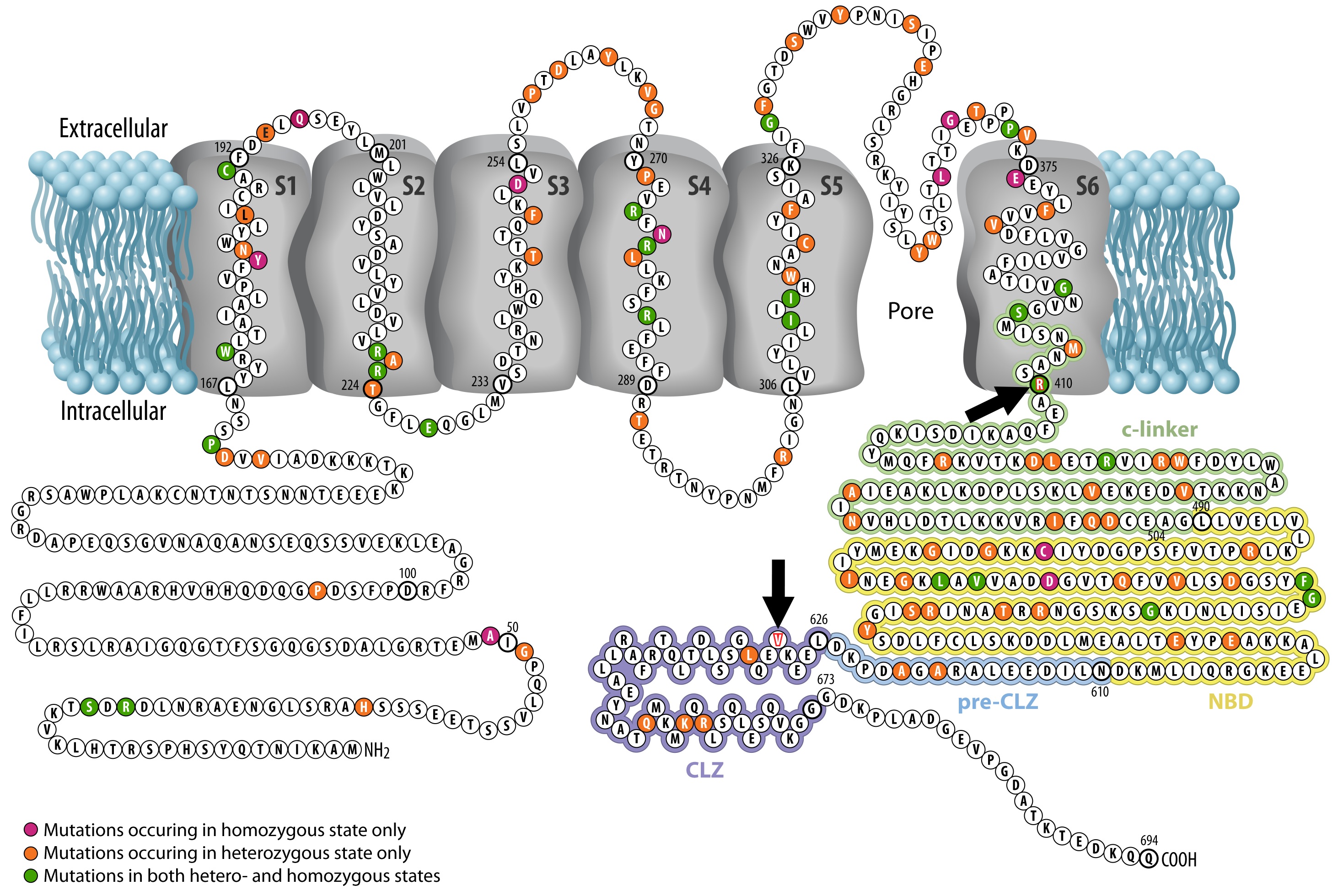

Supplement: S2 Fig — Schematic representation of human CNGA3 molecule deduced from multiple sequence alignments. Canine R424 residue affected in German shepherd with achromatopsia is located at the terminal cytoplasmic end of S6 (arrow) and corresponds to R410 of the human CNGA3. The V644 mutated in Labrador retriever is positioned within CLZ domain and corresponds to hCNGA3-V630 (arrow). All known human disease-associated mutations are denoted and color-coded. Mutations in homo-, hetero- as well as both, homo- and heterozygous states are scattered throughout all domains with mutational hotspot located in the C-terminal part of the protein. See also S3 Table) for additional details. (TIFF) [file pone.0138943.s002.tiff]

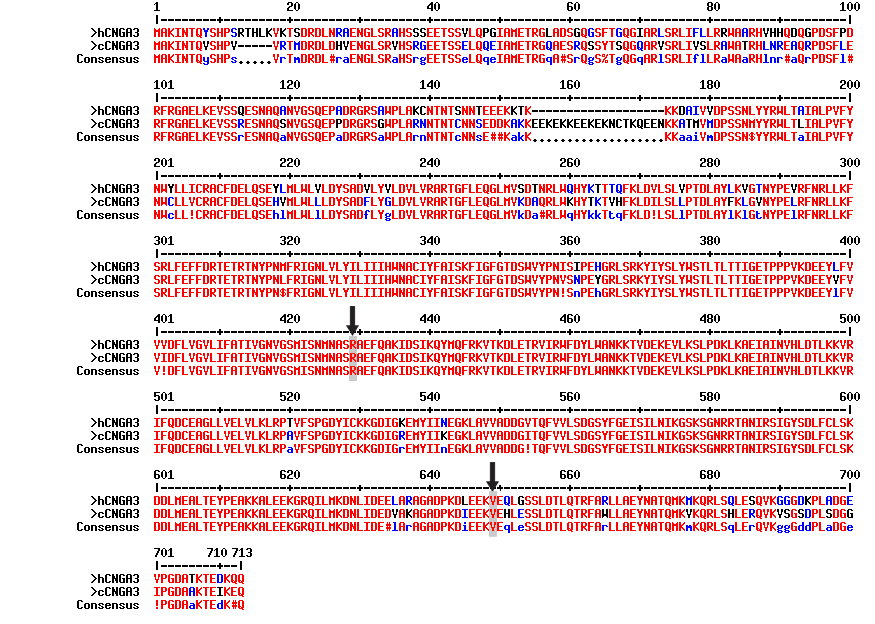

Supplement: S3 Fig — Orthologs of human (NCBI# NP_001289.1) and canine (NCBI# NP_001288041.1) CNGA3 demonstrate >82% of amino acid sequence identity with the highest degree of conservation in the C-terminal half, a region affected by the two novel canine mutations (arrows). Evolutionarily conserved residues are highlighted in red. h = human, c = canine; Sequence alignment was prepared using Mutlalin v.5.4.1. (TIFF) [file pone.0138943.s003.tiff]
