# Supplementary material for: Canine CNGA3 Gene Mutations Provide Novel Insights into Human Achromatopsia-Associated Channelopathies and Treatment
Source: PLoS One. 2015 Sep 25;10(9):e0138943. doi: 10.1371/journal.pone.0138943 (PMC4583268; doi:10.1371/journal.pone.0138943)
Supplement: S1 Table — (DOCX) [file pone.0138943.s006.docx]

**Table S1**

**List of primers and PCR conditions used for canine *CNGA3* gene analysis**

| **PRIMER** | **NUCLEOTIDE SEQUENCE 5’- 3’ (Forward Primer/Reverse Primer)** | **Annealing temp.** (**°C**) | **Amplicon size** (**bp**) |
| --- | --- | --- | --- |
| **5’UTR** | TGCAATGTAGCTGGAACTATGG**/**TGCAGAGAGTCCAATGAATGTC | 58° | 817 |
| **Exon 1** | TGCAATGTAGCTGGAACTATGG**/**AGGAGCACTGCGAATAGTAAGC | 60° | 395 |
| **Exon 2** | GTACATACGGTCTGGCCTTAGC**/**TTTCTCAGCTCAGGACACTTGA | 60° | 727 |
| **Exon 3** | TCGTACCTGGGTACTTGTTCCT**/**CTGTGAGAGCATGCAGTATTCC | 60° | 756 |
| **Exon 4** | CCAAATGTTCATCCTCTCTTCC**/**CATAAAGGAACTCCGATTTTGC | 58° | 594 |
| **Exon 5a** | CATACCCTCTCCTCCTTGTGAC**/**GGTGTGGAATGAAGTTTGTGAA | 62° | 535 |
| **Exon 5b** | CGTGCTATGCTGCAATTTACTC**/**CCTCCTCAGAGATGGAAAAATG | 60° | 586 |
| **Exon 6** | ATTGTCTGCATGAGAAATGGTG**/**CCATTGTAGAGGGAGCAGTACC | 58° | 705 |
| **Exon 7_1** | GCTGCCCTAACTATGCTTTCTT**/**CTCTTCAGCACCTCCTTCTCAT | 60° | 816 |
| **Exon 7_2** | GATCCGGTGGTTTGACTACCT**/**GTAAGGAGAATGCGAATGCAAC | 60° | 884 |
| **3’UTR** | AAGATCAAACCCGTAGGCTGTA**/**CATACTTGAAAATGCAGCACCT | 56° | 712 |
| **cDNA** | GGACTGAACTTGACAAAACAAGATG**/**ACACAGACCTCTGCATTTCACTG | 62° | 2166 |
