# Supplementary material for: Canine CNGA3 Gene Mutations Provide Novel Insights into Human Achromatopsia-Associated Channelopathies and Treatment
Source: PLoS One. 2015 Sep 25;10(9):e0138943. doi: 10.1371/journal.pone.0138943 (PMC4583268; doi:10.1371/journal.pone.0138943)
Supplement: S2 Table — (DOCX) [file pone.0138943.s007.docx]

**Table S2**

**Summary of cyclic nucleotide**-**activated** **conductance recorded for CNGA3-WT and a set of R424- and E306-mutant channels**

| **PARAMETERS** | **CNGA3-WT** | **R424W** | **R424K** | **R424E** | **E306D** | **E306R** | **E306R-R424E** |
| --- | --- | --- | --- | --- | --- | --- | --- |
| **cGMP** | 100% | 0% | 53% | 0% | 80% | 0% | 100% |
| ^(a)^**Mean I_cGMP_ (**pA**)** | - 374.5 ± 77.7 | NR | - 49.0 ± 14.1 | NR | - 250.05 ± 111.8 | NR | - 430.4 ± 60.3 |
| **cAMP** | 100% | 0% | 0% | 0% | 80% | 0% | 0% |
| ^(b)^**I_cAMP_ / I_cGMP_** | 0.21 ± 0.02 | - | - | - | 0.29 ± 0.03 | - | - |
| ^(c)^**cGMP K_0.5_ (**µM**)** | 12.3 ± 1.6 | - | - | - | - | - | 101.9 ± 8.6 |

^(a)^Mean cGMP-activated currents at -60mV ± SEM (n ≥ 10); ^(b)^I_cAMP_/I_cGMP_ efficacy ± SEM; ^(c)^cGMP
dose-response measurements ± SEM; NR = non-responsive;
