# Supplementary material for: Canine CNGA3 Gene Mutations Provide Novel Insights into Human Achromatopsia-Associated Channelopathies and Treatment
Source: PLoS One. 2015 Sep 25;10(9):e0138943. doi: 10.1371/journal.pone.0138943 (PMC4583268; doi:10.1371/journal.pone.0138943)
Supplement: S3 Table — (DOCX) [file pone.0138943.s008.docx]

**Table S3**

**A comprehensive summary of human *CNGA3* variants and their phenotypic consequences**

| **ΔAA (1)** | **ΔAA (2)** | **ΔcDNA (1)** | **ΔcDNA (2)** | **Diagnosis** | **Photopic ERG** | **References** |
| --- | --- | --- | --- | --- | --- | --- |
| S21X | S21X | C62G | C62G | ACHR | Severely Reduced | Li *et al.* (2014) |
|  | V529M | C62G | G1585A | ACHR | Severely Reduced | Li *et al.* (2014) |
|  | E590K | C62G | G1768A | CORD | Extinguished | Li *et al.* (2014) |
|  |  | C62G | G1768A | ACHR | Severely Reduced | Li *et al.* (2014) |
| R23X | R23X | C67T | C67T | ACHR | Extinguished | Johnson *et al.* (2004) |
|  | D260N | C67T | G778A | CORD | Extinguished | Li *et al.* (2014) |
|  | R427C | NL | NL | CD | Not Available | Koeppen *et al.* (2008) |
|  | F547L | NL | C1641A | cACHR | Extinguished | Reuter *et al.* (2008) |
| H36RfsX118 | R427C | 107-110del | C1279T | ACHR | Extinguished | Thomas *et al.* (2012) |
| G49fs | W316X | 148insG | G947A | NC | Not Available | Wissinger *et al.* (2001) |
| A51fs15 | A51fs15 | 130_151dup22 | 130_151dup22 | ACHR | Extinguished | Zelinger *et al.* (2015) |
| P95L | ND | C284T | ND | CD | Not Available | Thiadens *et al.* (2010) |
| V160G | F547L | NL | C1641A | cACHR | Extinguished | Reuter *et al.* (2008) |
| D162V | D260N | A485T | G778A | cACHR | Extinguished | Wissinger *et al.* (2001) |
|  |  | A485T | G778A | ACHR | Not Available | Thiadens *et al.* (2009) |
| P163L | P163L | C528T | C528T | RM | Not Available | Kohl *et al.* (1998) |
|  | R283W | C488T | C847T | ACHR | Extinguished | Varsányi *et al.* (2005) |
|  | R436W | C488T | C1306T | cACHR | Extinguished | Wissinger *et al.* (2001) |
| W171X | W171X | G512A | G512A | CORD | Mildly Reduced | Li *et al.* (2014) |
| W171C | R221X | G513T | C661T | ACHR | Extinguished | Li *et al.* (2014) |
|  | R283W | G513T | C847T | CORD | Extinguished | Li *et al.* (2014) |
| Y181C | Y181C | A542G | A542G | iACHR | Reduced | Wissinger *et al.* (2001) |
| N182Y | F547L | A544T | C1641A | cACHR | Extinguished | Wissinger *et al.* (2001) |
| L186F | R277C | C556T | C829T | cACHR | Extinguished | Wissinger *et al.* (2001) |
| C191Y | C191Y | G572A | G572A | iACHR | Residual | Wissinger *et al.* (2001) |
|  | R277C | G572A | C829T | cACHR | Extinguished | Wissinger *et al.* (2001) |
| E194K | F547L | G580A | C1641A | cACHR | Extinguished | Wissinger *et al.* (2001) |
| Q196X | Q196X | C586T | C586T | ACHR | Extinguished | Johnson *et al.* (2004) |
| R221X | R221X | C661T | C661T | ACHR | Extinguished | Johnson *et al.* (2004) |
|  | R277C | C661T | C829T | CORD | Severely Reduced | Li *et al.* (2014) |
|  | V529M | C661T | G1585A | CORD | Severely Reduced | Li *et al.* (2014) |
| A222P | G557R | NL | NL | iACHR | Reduced | Reuter *et al.* (2008) |
| R223Q | R436W | G668A | C1306T | ACHR | Extinguished | Li *et al.* (2014) |
| R223W | ND | C667T | ND | cACHR | Extinguished | Johnson *et al.* (2004) |
|  | ND | NL | ND | ACHR | Extinguished | Wiszniewski *et al.* (2007) |
|  | R223W | C667T | C667T | NC | Not Available | Wissinger *et al.* (2001) |
|  |  | C667T | C667T | CORD | Severely Reduced | Li *et al.* (2014) |
|  | S341P | C667T | T1021C | ACHR | Extinguished | Nishiguchi *et al.* (2005) |
|  |  | C667T | T1021C | iACHR | Residual | Wissinger *et al.* (2001) |
|  | R410W | C667T | C1228T | ACHR | Extinguished | Nishiguchi *et al.* (2005) |
|  | R427C | NL | NL | ACHR | Not Available | Koeppen *et al.* (2008) |
|  | E590K | C667T | G1768A | ACHR | Extinguished | Nishiguchi *et al.* (2005) |
| R223G | G557R | NL | NL | ACHR | Extinguished | Wiszniewski *et al.* (2007) |
| T224I | SSC | C671T | 674-2A>C | CORD | Moderately Reduced | Li *et al.* (2014) |
|  | R283W | C671T | C847T | CORD | Severely Reduced | Li *et al.* (2014) |
| T224R | T369S | C671G | C1106G | iACHR | Reduced | Wissinger *et al.* (2001) |
| E228K | E228K | G682A | G682A | iACHR | Reduced | Reuter *et al.* (2008) |
|  | V266M | G682A | G796A | CD* | Extinguished | Thiadens *et al.* (2010) |
| T245M | T245M | NL | NL | ACHR | Extinguished | Johnson *et al.* (2004) |
| F249S | E344X | T746C | G1030T | ACHR | Extinguished | Nishiguchi *et al.* (2005) |
| D252N | D252N | G754A | G754A | ACHR | Not Available | Koeppen *et al.* (2008) |
| P258R | V373Rfs4 | C773G | 1116dup | CORD | Extinguished | Li *et al.* (2014) |
| NL | R277C | G778A | C829T | ACHR | Extinguished | Zelinger *et al.* (2015) |
| Y263D | T565M | T787G | C1694T | ACHR | Severely Reduced | Nishiguchi *et al.* (2005) |
| G267D | Q655X | G800A | C1963T | cACHR | Extinguished | Wissinger *et al.* (2001) |
| P271A | G329C | C811G | G985T | ACHR | Extinguished | Zelinger *et al.* (2015) |
| R274K | L278P | G821A | T833C | ACHR | Extinguished | Li et al. (2014) |
| R274S | R274S | G822T | G822T | ACHR | Residual | Azam *et al.* (2010) |
| N276S | N276S | A827G | A827G | ACHR | Diminished | Saqib *et al.* (2011) |
| R277C | R277C | C829T | C829T | ACHR | Extinguished | Nishiguchi *et al.* (2005) |
|  | R283W | C829T | C847T | CD | Extinguished | Wissinger *et al.* (2001) |
|  | G329C | C829T | G985T | ACHR | Extinguished | Zelinger *et al.* (2015) |
|  | R427C | C829T | C1279T | iACHR | Residual | Wissinger *et al.* (2001) |
|  | R436W | C829T | C1306T | NC | Not Available | Wissinger *et al.* (2001) |
|  | R439W | NL | C1315T | cACHR | Extinguished | Reuter *et al.* (2008) |
|  | L527R | C829T | T1580G | ACHR | Extinguished | Lam *et al.* (2011) |
|  | Q537X | C829T | C1609T | NC | Extinguished | Wissinger *et al.* (2001) |
|  | F547L | C829T | C1641A | cACHR | Extinguished | Wissinger *et al.* (2001) |
|  |  | NL | C1641A | cACHR | Extinguished | Reuter *et al.* (2008) |
|  | A469T | NL | G1405A | iACHR/OCT | Extinguished | Reuter *et al.* (2008) |
|  | G557R | C829T | G1669A | iACHR | Extinguished | Wissinger *et al.* (2001) |
|  | R563C | NL | C1687T | iACHR | Not Available | Koeppen *et al.* (2008) |
|  | Y573C | C829T | A1718G | ACHR | Not Available | Thiadens *et al.* (2009) |
|  |  | C829T | A1718G | cACHR | Extinguished | Wissinger *et al.* (2001) |
| R277G | S341P | NL | NL | cACHR | Not Available | Koeppen *et al.* (2010) |
| R277H | R277H | G830A | G830A | cACHR | Extinguished | Wissinger *et al.* (2001) |
|  |  | G830A | G830A | ACHR | Extinguished | Li *et al.* (2014) |
|  | R283Q | NL | G848A | CD | Extinguished | Reuter *et al.* (2008) |
|  | W358X | G830A | G1074A | CORD | Severely Reduced | Li *et al.* (2014) |
|  | R439W | G830A | C1315T | ACHR | Extinguished | Li *et al.* (2014) |
|  | V529M | G830A | G1585A | CORD | Extinguished | Li *et al.* (2014) |
|  | G557R | G830A | G1669A | ACHR | Not Available | Zelinger *et al.* (2015) |
| L278P | R436W | T833C | C1306T | ACHR | Severely Reduced | Li *et al.* (2014) |
| R283W | R283W | C847T | C847T | ACHR | Extinguished | Varsányi *et al.* (2005) |
|  |  | C847T | C847T | ACHR | Not Available | Ahuja *et al.* (2008) |
|  |  | C887T | C887T | RM | Not Available | Kohl *et al.* (1998) |
|  |  | C847T | C847T | NC | Extinguished | Wissinger *et al.* (2001) |
|  |  | C847T | C847T | NC | Not Available | Wissinger *et al.* (2001) |
|  |  | C847T | C847T | iACHR | Strongly Reduced | Wissinger *et al.* (2001) |
|  |  | C847T | C847T | iACHR | Residual | Wissinger *et al.* (2001) |
|  |  | C847T | C847T | cACHR | Extinguished | Wissinger *et al.* (2001) |
|  | P372S | C847T | C1114T | cACHR | Extinguished | Wissinger *et al.* (2001) |
|  | F380S | C847T | T1139C | cACHR | Extinguished | Wissinger et al. (2001) |
|  | G397V | C847T | G1190T | ACHR | Not Available | Ahuja *et al.* (2008) |
|  | R427C | NL | NL | ACHR | Reduced | Koeppen *et al.* (2008) |
|  | F547L | C847T | C1641T | ACHR | Extinguished | Varsányi *et al.* (2005) |
|  |  | NL | C1641A | cACHR | Extinguished | Reuter *et al.* (2008) |
|  |  | NL | C1641A | ACHR | Extinguished | Reuter *et al.* (2008) |
|  |  | NL | C1641A | ACHR | Not Available | Reuter *et al.* (2008) |
|  | R563H | C847T | G1688A | iACHR | Not Available | Wissinger *et al.* (2001) |
|  | T565M | C847T | C1694T | iACHR | Responsive | Wissinger *et al.* (2001) |
| R283Q | ND | G848A | ND | NC | Extinguished | Wissinger *et al.* (2001) |
|  | R427C | G848A | C1279T | ACHR | Extinguished | Doucette *et al.* (2013) |
|  | L433W | G848A | T1298G | ACHR | Not Available | Koeppen et al. (2008) |
|  | L433W | G848A | NL | cACHR | Extinguished | Reuter *et al.* (2008) |
|  | R436W | G848A | C1306T | ACHR | Extinguished | Zelinger *et al.* (2015) |
|  | R436W | G848A | NL | cACHR | Extinguished | Reuter *et al.* (2008) |
|  | G557R | G848A | G1669A | ACHR | Not Available | Zelinger *et al.* (2015) |
|  | G557R | G848A | G1669A | NC | Not Available | Wissinger et al. (2001) |
|  | G557R | G888A | G1709A | RM | Not Available | Kohl *et al.* (1998) |
|  | R569H | G848A | NL | cACHR | Extinguished | Reuter *et al.* (2008) |
| T291R | F547L | C912G | C1681A | RM | Not Available | Kohl *et al.* (1998) |
| T291Rfs77 | V529M | 872_873del | G1585A | CORD | Severely Reduced | Li *et al.* (2014) |
|  | K659X | 872_873del | A1975T | CORD | Not Available | Li *et al.* (2014) |
| R302G | V529M | A904G | G1585A | ACHR | Not Available | Zelinger *et al.* (2015) |
|  | F547C | A904G | T1640G | ACHR | Reduced | Zelinger *et al.* (2015) |
|  |  | A904G | T1640G | ACHR | Not Available | Zelinger *et al.* (2015) |
| I312del | I312del | 934-936delATC | 934-936delATC | cACHR | Extinguished | Wissinger *et al.* (2001) |
|  | R427C | NL | NL | ACHR | Not Available | Koeppen *et al.* (2008) |
| I314del | I314del | 940-942delATC | 940-942delATC | ACHR | Extinguished | Zelinger *et al.* (2015) |
|  |  | 940-942delATC | 940-942delATC | ACHR | Not Available | Zelinger *et al.* (2015) |
|  | NL | 940-942delATC | A1454T | ACHR | Extinguished | Zelinger *et al.* (2015) |
|  | V529M | 940-942delATC | G1585A | ACHR | Extinguished | Zelinger *et al.* (2015) |
|  | F547C | 940-942delATC | T1640G | ACHR | Extinguished | Zelinger *et al.* (2015) |
| C319fsX | F547L | NL | C1641A | ACHR | Not Available | Reuter *et al.* (2008) |
| F322S/R436Q | W440X | T965C/G1307A | G1319A | ACHR | Severely Reduced | Li *et al.* (2014) |
| G329C | G329C | G985T | G985T | ACHR | Extinguished | Zelinger *et al.* (2015) |
|  |  | G985T | G985T | ACHR | Not Available | Zelinger *et al.* (2015) |
| F330S | R439W | T989C | C1315T | CORD | Severely Reduced | Li *et al.* (2014) |
|  | V529M | T989C | G1585A | CORD | Severely Reduced | Li *et al.* (2014) |
| S334F | R439W | C1001T | C1315T | CORD | Extinguished | Li *et al.* (2014) |
| Y337Sfs28 | D543_S545del | 1010_1012del/ insCAATCCCAGTG | 1627_1635del | ACHR | Extinguished | Li *et al.* (2014) |
| E344X | R436W | G1030T | C1306T | ACHR | Extinguished | Nishiguchi *et al.* (2005) |
| Y357C | T565M | A1070G | C1694T | OCT | Severely Reduced | Vincent *et al.* (2011) |
| W358X | R439W | G1074A | C1315T | ACHR | Extinguished | Li *et al.* (2014) |
|  | V529M | G1074A | G1585A | CORD | Extinguished | Li *et al.* (2014) |
|  | E590K | G1074A | G1768A | ACHR | Extinguished | Li *et al.* (2014) |
|  | SSC | G1074A | C396-11G | ACHR | Extinguished | Li *et al.* (2014) |
|  |  | G1074A | C396-11G | CORD | Severely Reduced | Li *et al.* (2014) |
| L363P | L363P | T1088C | T1088C | ACHR | Residual | Koeppen *et al.* (2010) |
| G367V | G367V | G1100T | G1100T | cACHR | Extinguished | Koeppen *et al.* (2010) |
|  |  | G1100T | G1100T | ACHR | Extinguished | Koeppen *et al.* (2010) |
|  |  | G1100T | G1100T | ACHR | Not Available | Koeppen *et al.* (2010) |
| P372S | P372S | C1114T | C1114T | ACHR | Residual | Koeppen et al. (2010) |
|  |  | C1114T | C1114T | cACHR | Extinguished | Koeppen et al. (2010) |
|  |  | C1114T | C1114T | NC | Not Available | Wissinger *et al.* (2001) |
| NL | NL | C1114T | C1981A | ACHR | Extinguished | Zelinger *et al.* (2015) |
| E376K | E376K | G1126A | G1126A | ACHR | Not Available | Koeppen *et al.* (2010) |
| NL | NL | G1126A | G1126A | ACHR | Extinguished | Zelinger *et al.* (2015) |
| V383Rfs36 | V529M | 1146dup | G1585A | ACHR | Moderately Reduced | Li *et al.* (2014) |
| G397V | G397V | G1190T | G1190T | ACHR | Not Available | Ahuja *et al.* (2008) |
| S401P | S401P | T1201C | T1201C | ACHR | Extinguished | Nishiguchi *et al.* (2005) |
|  | R427C | T1201C | C1279T | ACHR | Severely Reduced | Nishiguchi *et al.* (2005) |
| M406T | ND | T1217C | ND | cACHR | Extinguished | Wissinger *et al.* (2001) |
| R410W | R410W | C1228T | C1228T | cACHR | Extinguished | Wissinger *et al.* (2001) |
| R410W | V529M | C1268T | G1625A | RM | Extinguished | Kohl *et al.* (1998) |
| R427C | R436W | C1279T | C1306T | iACHR | Severely Reduced | Wissinger *et al.* (2001) |
|  | V451fs | C1279T | 1350insG | iACHR | Extinguished | Wissinger *et al.* (2001) |
|  | L527R | C1279T | T1580G | ACHR | Reduced | Doucette *et al.* (2013) |
|  | R569H | C1279T | G1706A | ACHR | Extinguished | Thomas *et al.* (2012) |
| D432fs32 | V529M | 1294delG | G1585A | ACHR | Extinguished | Zelinger *et al.* (2015) |
| R436W | ND | C1306T | ND | ACHR** | Extinguished | Johnson *et al.* (2004) |
|  | ND | C1306T | ND | iACHR | Residual | Wissinger *et al.* (2001) |
|  | R436W | C1306T | C1306T | CORD | Extinguished | Li *et al.* (2014) |
|  | R499X | C1306T | C1495T | CORD | Extinguished | Li *et al.* (2014) |
|  | F547L | C1306T | C1641A | cACHR | Extinguished | Wissinger *et al.* (2001) |
|  | R563H | C1306T | G1688A | iACHR | Severely Reduced | Wissinger *et al.* (2001) |
|  | L633P | C1306T | T1898C | ACHR | Severely Reduced | Goto-Omoto *et al.* (2006) |
| R439W | D543_S545del | C1315T | 1627_1635del | ACHR | Extinguished | Li *et al.* (2014) |
| W440X | G516E | G1320A | G1547A | cACHR | Extinguished | Wissinger *et al.* (2001) |
| V456fsX | G557R | NL | NL | OCT | Extinguished | Reuter *et al.* (2008) |
| N471S | ND | A1412G | ND | CD | Reduced | Wissinger *et al.* (2001) |
| I482fs | R569H | 1443insC | G1706A | ACHR | Extinguished | Johnson *et al.* (2004) |
| R499X | V529M | C1495T | G1585A | ACHR | Extinguished | Li *et al.* (2014) |
|  | SSC | C1495T | C396-11G | CORD | Extinguished | Li *et al.* (2014) |
| C510S | C510S | G1529C | G1529C | cACHR | Extinguished | Wissinger *et al.* (2001) |
| G513E | R569H | G1538A | G1706A | cACHR | Extinguished | Wissinger *et al.* (2001) |
| I522T | F547L | T1565C | C1641A | iACHR | Not Available | Wissinger *et al.* (2001) |
|  |  | T1565C | C1641A | ACHR | Not Available | Thiadens *et al.* (2009) |
| G525D | T565M | G1574A | C1694T | iACHR | Residual | Wissinger *et al.* (2001) |
| L527R | L527R | T1580G | T1580G | ACHR | Extinguished | Doucette *et al.* (2013) |
| L527M | L527M | C1579A | C1579A | LCA | Extinguished | Wang *et al.* (2011) |
| V529M | V529M | G1585A | G1585A | CORD | Severely Reduced | Li *et al.* (2014) |
|  |  | G1585A | G1585A | ACHR | Not Available | Zelinger *et al.* (2010) |
|  |  | G1585A | G1585A | ACHR | Extinguished | Nishiguchi *et al.* (2005) |
|  | F547C | G1585A | T1640G | ACHR | Extinguished | Zelinger *et al.* (2015) |
|  | G548R | G1585A | G1642A | ACHR | Not Available | Zelinger *et al.* (2015) |
|  | S570N | G1585A | G1709A | CORD | Moderately Reduced | Li *et al.* (2014) |
|  | E590K | G1585A | G1768A | ACHR | Severely Reduced | Nishiguchi *et al.* (2005) |
| D533H | D533H | G1597C | G1597C | CORD | Extinguished | Li *et al.* (2014) |
| V540I | ND | G1618A | ND | CD | Not Available | Thiadens *et al.* (2010) |
| F547L | ND | C1641A | ND | cACHR | Extinguished | Wissinger *et al.* (2001) |
|  | F547L | C1641A | C1641A | cACHR | Extinguished | Wissinger *et al.* (2001) |
|  |  | C1641A | C1641A | ACHR | Extinguished | Johnson *et al.* (2004) |
|  |  | C1641T | C1641T | ACHR | Extinguished | Varsányi *et al.* (2005) |
|  |  | C1641A | C1641A | ACHR | Extinguished | Thomas *et al.* (2012) |
|  |  | C1641A | C1641A | cACHR | Extinguished | Reuter *et al.* (2008) |
|  |  | C1641A | C1641A | ACHR | Extinguished | Reuter *et al.* (2008) |
|  |  | C1641A | C1641A | ACHR | Reduced | Reuter *et al.* (2008) |
|  |  | C1641A | C1641A | ACHR | Not Available | Reuter *et al.* (2008) |
|  | R563H | C1641A | G1688A | CD | Extinguished | Wissinger *et al.* (2001) |
| G548R | G548R | G1642A | G1642A | ACHR | Extinguished | Johnson *et al.* (2004) |
| G557R | G557R | G1669A | G1669A | ACHR | Extinguished | Zelinger *et al.* (2015) |
| T565M | ND | C1694T | ND | CD | Not Available | Thiadens *et al.* (2010) |
| E590K | ND | G1768A | ND | ACHR | Extinguished | Nishiguchi *et al.* (2005) |
| E593K | ND | G1777A | ND | iACHR | Extinguished | Wissinger *et al.* (2001) |
| A619V | ND | C1856T | ND | CD | Not Available | Thiadens *et al.* (2010) |
| A621E | ND | NL | ND | ACHR | Extinguished | Wiszniewski *et al.* (2007) |

ACHR: Achromatopsia (unclassified type);

iACHR: Incomplete Achromatopsia;

cACHR: Complete Achromatopsia;

CORD: Cone-Rod Dystrophy;

CD: Cone Dystrophy;

RM: Rod Monochromacy;

OCT: Oligocone Trichromacy;

LCA: Leber Congenital Amaurosis;

NC: Not Classified;

SSC: Splice Site Changed;

NL: Nucleotide or Amino Acid change not listed;

ND: Not Determined;

*Also homozygous for CNGB3 p.R403Q;

**Also heterozygous for CNGB3 p.N27S and a CNGB3 5'UTR (-36) mutation;

Unless otherwise noted, extinguished photopic ERG is <2μV; severely reduced photopic ERG is <10μV;

Note: each family is listed only once if multiple individuals are reported with identical phenotype.

**Table S3 references**

Ahuja, Y., Kohl, S., and Traboulsi, E.I. (2008). CNGA3 mutations in two United Arab Emirates families with achromatopsia. *Mol. Vis*., **14**, 1293-1297.

Azam, M., Collin, R.W., Shah, S.T., Shah, A.A., Khan, M.I., Hussain, A., Sadeque, A., Strom, T.M., Thiadens, A.A., Roosing, S.*, et al.* (2010). Novel CNGA3 and CNGB3 mutations in two Pakistani families with achromatopsia. *Mol. Vis*., **16**, 774-781.

Doucette, L., Green, J., Black, C., Schwartzentruber, J., Johnson, G.J., Galutira, D., and Young, T.L. (2013). Molecular genetics of achromatopsia in Newfoundland reveal genetic heterogeneity, founder effects and the first cases of Jalili syndrome in North America. *Ophthalmic Genet*., **34**, 119-129.

Goto-Omoto, S., Hayashi, T., Gekka, T., Kubo, A., Takeuchi, T., and Kitahara, K. (2006) Compound heterozygous CNGA3 mutations (R436W, L633P) in a Japanese patient with congenital achromatopsia. *Vis. Neurosci.*, **23**, 395-402.

Johnson, S., Michaelides, M., Aligianis, I.A., Ainsworth, J.R., Mollon, J.D., Maher, E.R., Moore, A.T., and Hunt, D.M. (2004). Achromatopsia caused by novel mutations in both CNGA3 and CNGB3. *J. Med. Genet.,* **41**, e20.

Koeppen, K., Reuter, P., Kohl, S., Baumann, B., Ladewig, T., and Wissinger, B. (2008). Functional analysis of human CNGA3 mutations associated with colour blindness suggests impaired surface expression of channel mutants A3(R427C) and A3(R563C). *Eur. J. Neurosci*., **27**, 2391-2401.

Koeppen, K., Reuter, P., Ladewig, T., Kohl, S., Baumann, B., Jacobson, S.G., Plomp, A.S., Hamel, C.P., Janecke, A.R., and Wissinger, B. (2010). Dissecting the pathogenic mechanisms of mutations in the pore region of the human cone photoreceptor cyclic nucleotide-gated channel. *Hum. Mutat*., **31**, 830-839.

Kohl, S.*,* Marx, T., Giddings, I., Jagle, H., Jacobson, S.G., Apfelstedt-Sylla, E., Zrenner, E., Sharpe, L.T., and Wissinger, B*.* (1998) Total colourblindness is caused by mutations in the gene encoding the alpha-subunit of the cone photoreceptor cGMP-gated cation channel. *Nat. Genet.,* **19**, 257-259.

Lam, K., Guo, H., Wilson, G.A., Kohl, S., and Wong, F. (2011). Identification of variants in CNGA3 as cause for achromatopsia by exome sequencing of a single patient. *Arch. Ophthalmol.,* **129**, 1212-1217.

Li, S., Huang, L., Xiao, X., Jia, X., Guo, X., and Zhang, Q. (2014). Identification of CNGA3 mutations in 46 families: common cause of achromatopsia and cone-rod dystrophies in Chinese patients. *JAMA Ophthalmol.,* **132**, 1076-1083.

Nishiguchi, K.M., Sandberg, M.A., Gorji, N., Berson, E.L., and Dryja, T.P. (2005) Cone cGMP-gated channel mutations and clinical findings in patients with achromatopsia, macular degeneration, and other hereditary cone diseases. *Hum. Mutat.,* **25**, 248-258.

Reuter, P., Koeppen, K., Ladewig, T., Kohl, S., Baumann, B., Wissinger, B., and Achromatopsia Clinical Study, G. (2008). Mutations in CNGA3 impair trafficking or function of cone cyclic nucleotide-gated channels, resulting in achromatopsia. *Hum. Mutat.,* **29**, 1228-1236.

Saqib, M.A., Awan, B.M., Sarfraz, M., Khan, M.N., Rashid, S., and Ansar, M. (2011). Genetic analysis of four Pakistani families with achromatopsia and a novel S4 motif mutation of CNGA3. *Jpn. J. Ophthal*., **55**, 676-680.

Thiadens, A.A., Slingerland, N.W., Roosing, S., van Schooneveld, M.J., van Lith-Verhoeven, J.J., van Moll-Ramirez, N., van den Born, L.I., Hoyng, C.B., Cremers, F.P., and Klaver, C.C. (2009). Genetic etiology and clinical consequences of complete and incomplete achromatopsia. *Ophthalmology,* **116**, 1984-1989 e1981.

Thiadens, A.A.*,* Roosing, S., Collin, R.W., van Moll-Ramirez, N., van Lith-Verhoeven, J.J., van Schooneveld, M.J., den Hollander, A.I., van den Born, L.I., Hoyng, C.B., Cremers, F.P., and Klaver, C.C*.* (2010) Comprehensive analysis of the achromatopsia genes CNGA3 and CNGB3 in progressive cone dystrophy. *Ophthalmology,* **117**, 825-830 e821.

Thomas, M.G., McLean, R.J., Kohl, S., Sheth, V., and Gottlob, I. (2012). Early signs of longitudinal progressive cone photoreceptor degeneration in achromatopsia. *Br. J. Ophthalmol*., **96**, 1232-1236.

Varsanyi, B., Wissinger, B., Kohl, S., Koeppen, K., and Farkas, A. (2005). Clinical and genetic features of Hungarian achromatopsia patients. *Mol. Vis*., **11**, 996-1001.

Vincent, A., Wright, T., Billingsley, G., Westall, C., and Heon, E. (2011). Oligocone trichromacy is part of the spectrum of CNGA3-related cone system disorders. *Ophthalmic Genet*., **32**, 107-113.

Wang, X., Wang, H., Cao, M., Li, Z., Chen, X., Patenia, C., Gore, A., Abboud, E.B., Al-Rajhi, A.A., Lewis, R.A.*, et al.* (2011). Whole-exome sequencing identifies ALMS1, IQCB1, CNGA3, and MYO7A mutations in patients with Leber congenital amaurosis. *Hum. Mutat*., **32**, 1450-1459.

Wissinger, B.*,* Gamer, D., Jagle, H., Giorda, R., Marx, T., Mayer S, Tippmann, S., Broghammer, M., Jurklies, B., Rosenberg, T*., et al.* (2001) CNGA3 mutations in hereditary cone photoreceptor disorders. *Am. J. Hum. Genet.,* **69**, 722-737.

Wiszniewski, W., Lewis, R.A., and Lupski, J.R. (2007). Achromatopsia: the CNGB3 p.T383fsX mutation results from a founder effect and is responsible for the visual phenotype in the original report of uniparental disomy 14. *Hum. Genet*., **121**, 433-439.

Zelinger, L., Greenberg, A., Kohl, S., Banin, E., and Sharon, D. (2010). An ancient autosomal haplotype bearing a rare achromatopsia-causing founder mutation is shared among Arab Muslims and Oriental Jews. *Hum. Genet*., **128**, 261-267.

Zelinger, L., Cideciyan, A.V., Kohl, S., Schwartz, S.B., Rosenmann, A., Eli, D., Sumaroka, A., Roman, A.J., Luo, X., Brown, C.*, et al.* (2015). Genetics and Disease Expression in the CNGA3 Form of Achromatopsia: Steps on the Path to Gene Therapy. *Ophthalmology*, **122**, 997-1007.
